# A core human gut microbe, *Mediterraneibacter gnavus*, produces a broad-spectrum bacteriocin mediterrocin

**DOI:** 10.1128/mbio.01523-25

**Published:** 2025-07-17

**Authors:** Gabrielle Mingolelli, Jessia Raherisoanjato, Duong Trinh, Yu Gao, Matthew Henke

**Affiliations:** 1 Department of Pharmaceutical Sciences, College of Pharmacy, University of Illinois Chicago 14681, Chicago, Illinois, USA; Carnegie Mellon University, Pittsburgh, Pennsylvania, USA

**Keywords:** bacteriocin, *Mediterraneibacter gnavus*, gut microbiome

## Abstract

A diverse, stable community of microbes in the human gut is strongly tied to health. The factors that regulate microbial community structure and dynamics are ill-defined. In this study, we discover mediterrocin, a bacteriocin produced by the core member of the human gut microbiota, *Mediterraneibacter gnavus* (previously *Ruminococcus gnavus*). Following a screen for antimicrobial production by clinical isolates of *M. gnavus*, mediterrocin was isolated by bioactivity-guided fractionation from strain RJX1121, identified by mass spectrometry, and confirmed by gene disruption. Mediterrocin, an 89 amino acid secreted protein, is a broad-spectrum bacteriocin. Annotation of the mediterrocin biosynthetic gene cluster revealed a wide distribution among Bacillota (previously Firmicutes), including the *M. gnavus* type strain ATCC249149. The type strain of *M. gnavus* also produces mediterrocin, but in lower titers than RJX1121 under our culture conditions. Mediterrocin is inhibitory to multiple *M. gnavus* strains, but with varying sensitivity, with isolates from patients with inflammatory bowel disease displaying the greatest susceptibility. Beyond *M. gnavus*, mediterrocin demonstrated inhibitory activity against other Lachnospiraceae and the gram-negative pathogen *Morganella morganii*. Further characterization of mediterrocin may clarify how this core member of the healthy microbiome interacts with other community members and how the relative abundance of *M. gnavus* changes in microbiome dysbiosis associated with several diseases.

**IMPORTANCE:**

*Mediterraneibacter gnavus* is a core member of the gut microbiome and implicated in several diseases. Direct competition between *M. gnavus* and other members of the microbiome may contribute to the shift from a diverse, structured community to dysbiosis observed in disease. Identifying molecular mechanisms of microbial competition will aid future studies of these disease-relevant bacteria and clarify how community structure is maintained. We report the discovery of mediterrocin, a bacteriocin present in many species in the orders Lachnospirales and Eubacteriales, but clade-specific within *M. gnavus*. Mediterrocin, produced by health-associated strains of *M. gnavus*, inhibits the growth of disease-associated *M. gnavus* strains and a broad spectrum of gut commensal bacteria and pathogens.

## INTRODUCTION

Loss of diversity and structure of the gut microbiome (dysbiosis) has been connected to multiple diseases, including inflammatory bowel disease (IBD) (1–3). It is not understood how this imbalance of commensal bacteria develops, but the imbalance likely results from a complex mixture of genetic, environmental, and microbial factors. Fecal microbiota transplantation (FMT) studies have shown that correcting the microbiome can treat diseases in which dysbiosis is implicated (4–6). By studying gut microbial interactions, we can identify factors that shape community composition, root causes of dysbiosis, and potential treatment strategies.

As community structure changes within the gut during dysbiosis, it can trigger inflammation of the surrounding and distant tissue (1). Microbiome structure is partly shaped by competition between bacteria, often through antimicrobial peptide or bacteriocin production. Bacteriocins provide the producing organisms a competitive advantage over bacteriocin-sensitive organisms. These bacteriocins can alter community structure through a variety of mechanisms. Bacteriocins made by resident bacteria can prevent colonization by incoming bacteria (7), or they can enable colonization by invading producers through niche clearing (7). Within a given species, bacteriocin genes are often strain specific, and they are rarely part of a species’ core genome (8). So, care should be taken not to assign an ecological role to an entire species based on bacteriocin production in a particular strain. Conversely, bacteriocins can be conserved across disparate species, in which case the bacteriocin may fulfill similar or different ecological functions, depending on other factors (e.g., biogeographical differences in producing organisms). Bacteriocins have been characterized from many gut microbiome members. Bacteroidetes produce diverse class IIa bacteriocins (bacteroidetocins) which target Bacteroidales outer membrane protein bamA (9, 10). Enterobacteriaceae produce class I and II bacteriocins (microcins) which narrowly target closely related species through diverse mechanisms (11, 12). Identifying factors underlying microbial competition, such as bacteriocins, particularly with a focus on strain-resolution of production, may provide mechanistic information on the establishment and development of microbial community structure.

We chose to look for bacteriocin production by *Mediterraneibacter [Ruminococcus] gnavus* (13), since *M. gnavus* is a core member of the healthy human gut microbiome (14) and has been associated with dysbiosis in a variety of diseases. Increases of specific strains of *M. gnavus* have been linked to symptom flares in IBD (15) and lupus (2, 16). Understanding how dysbiosis arises requires more thorough study of disease-relevant *M. gnavus* strains. While *M. gnavus* E1, isolated from a healthy adult, produces two bacteriocins, ruminococcins A and C (17, 18), the ruminococcins have a limited distribution in *M. gnavus* and are not found in the type strain or a series of clinical isolates from patients with IBD. In our study, we focused on screening for competition via antimicrobial production between these IBD clinical isolates of *M. gnavus* in addition to the type strain ATCC29149 and strain ATCC35913, which were isolated from healthy adult feces. Identifying molecular mechanisms of competition between disease-relevant *M. gnavus* strains will allow for the study of differential strain abundance in dysbiotic gut microbiomes.

This study presents the purification and characterization of mediterrocin, a bacteriocin produced by *M. gnavus* strains RJX1121, RJX1123, and ATCC29149. Mediterrocin is synthesized as a 114-residue protein containing a 25-residue signal sequence, which is cleaved upon secretion from the cell to yield an 89-residue mature protein. Mediterrocin was isolated by bioactivity-guided fractionation and identified by liquid chromatography-tandem mass spectrometry. We confirmed that mediterrocin is responsible for antimicrobial activity through the generation of the mediterrocin disruption mutant in strain RJX1121. Analysis of the biosynthetic gene cluster (BGC) revealed a wide distribution of mediterrocin in Bacillota [Firmicutes] as well as conservation in the type strain of *M. gnavus* ATCC29149. Mediterrocin is inhibitory to multiple *M. gnavus* strains but has varying sensitivity, with isolates from patients with IBD displaying the greatest susceptibility. Producer strains are resistant to mediterrocin, likely due to a putative immunity protein within the bacteriocin BGC. In a screen of gut bacteria, mediterrocin demonstrated modest inhibition against closely related Lachnospiraceae and the gram-negative pathogen *Morganella morganii*. Future characterization of mediterrocin will explore the ecological role of producer and sensitive strains, particularly as it relates to disease dysbiosis.

## RESULTS

### Purification of mediterrocin from *M. gnavus* RJX1121 culture

To identify antimicrobials produced by *M. gnavus* which could contribute to inter-strain competition in the gut microbiome, we screened the cell-free supernatant (CFS) from 10 isolates of *M. gnavus* for the ability to inhibit the growth of another *M. gnavus* strain. We found that *M. gnavus* RJX1121 CFS inhibited the growth of several other *M. gnavus* strains (RJX1119, RJX1124, RJX1125, and RJX1128) (Table S1). The antimicrobial did not flow through a 10 kD spin filter, and activity diminished following heat treatment (Fig. S1A). This combination of large molecular weight and bioactivity loss after boiling suggested that a bacteriocin was responsible for the activity. Purification of mediterrocin was achieved with strong anion exchange chromatography (SAX) followed by size exclusion chromatography (SEC) (Fig. S1B). Bioactivity-guided fractionation allowed for the identification of mediterrocin in the active subfraction following SEC. This subfraction is not pure mediterrocin, and at least two other proteins were observed by LC-MS/MS; however, the major protein in the subfraction is mediterrocin, and the other proteins were too low in abundance for identification by MS/MS guided sequence annotation. No protein bands, including mediterrocin, were visible on an SDS-PAGE gel, which is less sensitive than LC-MS detection (Fig. S1C).

### Mediterrocin has antimicrobial effects against select *M. gnavus* strains

**Fig 1:**
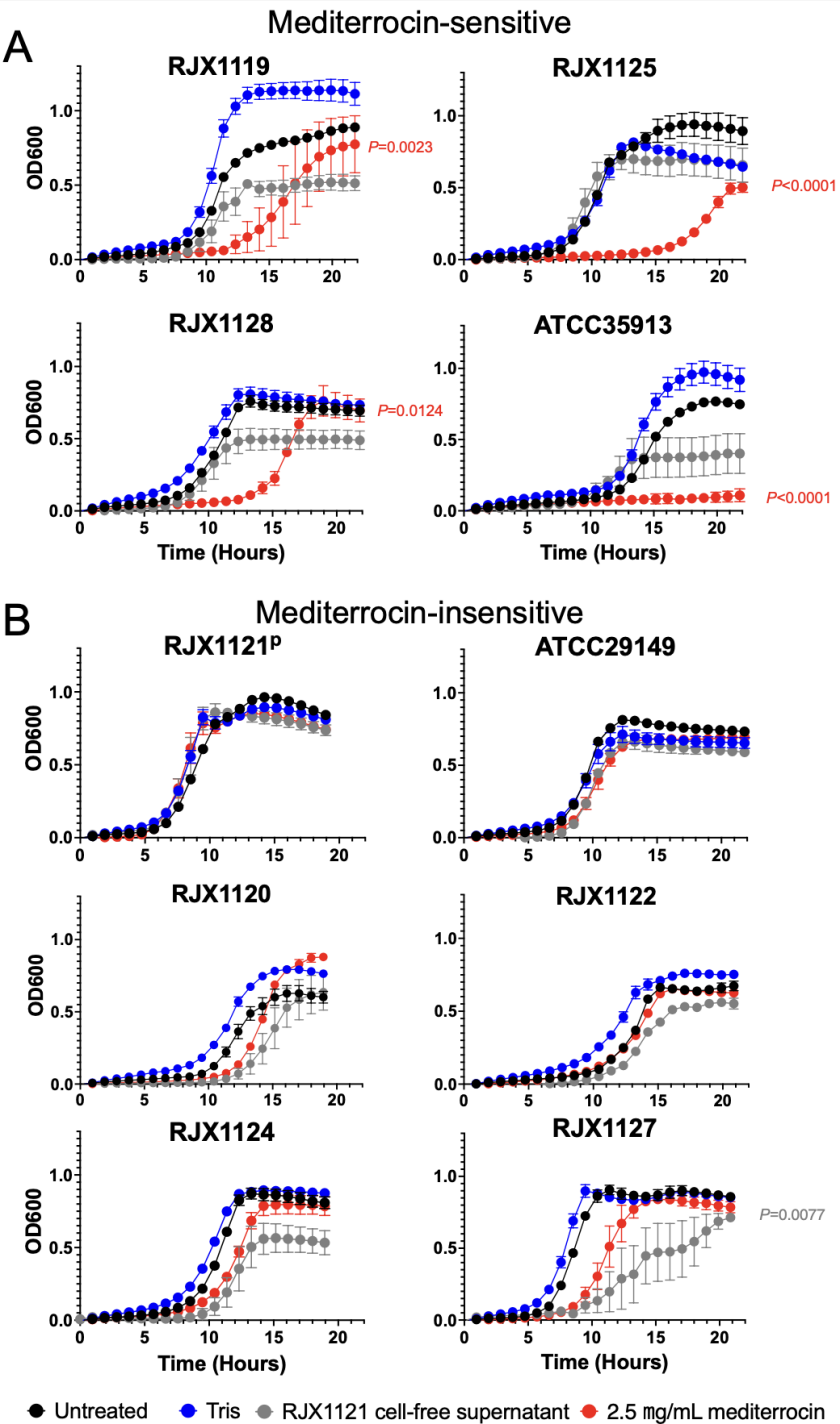
Mediterrocin inhibits the growth of select strains of *M. gnavus****.*** Growth curves of *M. gnavus* strains treated with *M. gnavus* RJX1121 CFS or 2.5 µg/mL mediterrocin in 20 mM Tris. (**A**) *M. gnavus* strains sensitive to mediterrocin and (**B**) strains insensitive to mediterrocin, including the mediterrocin producer strain RJX1121, which is marked with a superscript “P.” Strains were designated as sensitive to mediterrocin from a significant decrease in AUC of mediterrocin treatment compared to 20 mM Tris control, where significance was defined as *P* < 0.05. Sensitivity to *M. gnavus* RJX1121 CFS was defined as a significant decrease in AUC of CFS treatment compared to untreated, where significance was defined as *P* < 0.05. Error bars represent SEM.

The spectrum of activity of mediterrocin was determined using 10 strains of *M. gnavus*, including the type strain ATCC29149. The antimicrobial effect of purified mediterrocin was compared to the bioactivity of *M. gnavus* RJX1121 CFS. We defined sensitivity to mediterrocin as a significant decrease in the AUC of the treated growth curve compared to control. Four *M*. *gnavus* strains are sensitive to purified mediterrocin (2.5 µg/mL total protein) (Fig. 1A). Six strains, including the producer strain RJX1121, did not have significant sensitivity to mediterrocin treatment (Fig. 1B).

Not all *M. gnavus* strains are equally sensitive to mediterrocin (Fig. 1, Table 1). At a lower mediterrocin dose (1.25 µg/mL total protein), there is a dose-dependent increase in percent growth in mediterrocin-sensitive strains RJX1128 and ATCC35913 (Fig. S2). The most sensitive *M. gnavus* strains are RJX1125, RJX1128, and ATCC35913 (Table 1). *M. gnavus* RJX1127 did not have a significant decrease in growth curve AUC, but there was a significant change in percent growth with CFS and mediterrocin treatment at maximum optical density of the controls (Fig. S2). This is due to an extended lag time that did not reach statistical significance (Fig. 1B).

**TABLE 1:** *M. gnavus* strains have varying sensitivity to mediterrocin^*a*^

*M. gnavus* strain	MIC (µg/mL total protein)
RJX1119	2.5
RJX1125	2.5
RJX1127	2.5
RJX1128	1.25
ATCC35913	1.25
RJX1120	>2.5
RJX1122	>2.5
RJX1124	>2.5
RJX1121^p^	>2.5
ATCC29149	>2.5

^*a*^ Minimum inhibitory concentration (MIC) of purified mediterrocin against 10 strains of *M. gnavus*. Mediterrocin producer strains are marked with a superscript “P”.

The strain specificity of mediterrocin’s antimicrobial effect raises questions about the mechanism of action. It is unlikely that mediterrocin is responsible for the full antimicrobial activity observed in *M. gnavus* RJX1121 CFS, but it is partially responsible for the bioactivity because it can reproduce some of the inhibitory effects when purified. For example, RJX1127 is significantly inhibited by CFS, but not mediterrocin (Fig. 1B).

### Identification of mediterrocin by LC-MS/MS

In the active fraction following SAX (Fig. S1B), tryptic peptides of mediterrocin were identified by peptide-spectral matching (PSM) and annotation as a lactococcin 972 family bacteriocin (Table S2 and S3). Mediterrocin was assigned to the hypothetical protein gene CDL19_RS00990 in *M. gnavus* RJX1121 (WP_005608346.1). Using signal peptide predictors, we predicted that mediterrocin is synthesized as a 114-residue peptide containing an N-terminal 25-residue signal peptide (Fig. S3A and B). Further evidence supporting the antimicrobial activity of mediterrocin is the annotation as a lactococcin 972 family bacteriocin, despite only 18% identity to the core of lactococcin 972 (lcn972) (WP_011117133.1) (Fig. S3C). Despite low sequence similarity, the AlphaFold (19) predicted structure of mediterrocin is similar to lcn972 (Fig. S3D).

**Fig 2:**
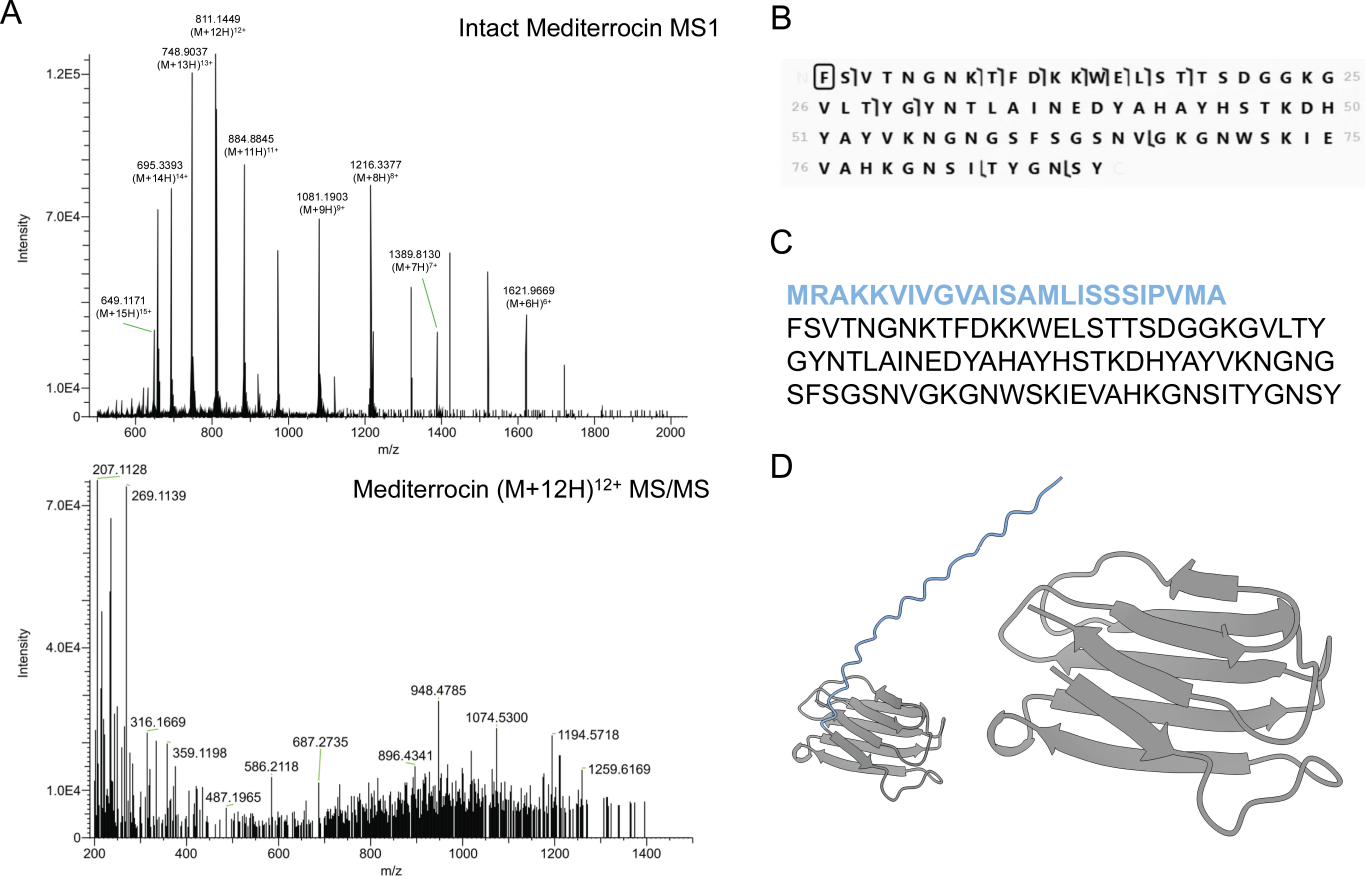
Identification of mediterrocin in size exclusion chromatography active subfraction by intact protein LC-MS/MS. (**A**) Mass spectra of mediterrocin purified from *M. gnavus* RJX1121 cell-free supernatant and MS/MS of precursor *m/z* 811.22 (M + 12H)^12+^ charge state of mediterrocin. (**B**) Mediterrocin MS/MS fragment map made with ProSight Lite with observed b ions represented by ך markers and *y* ions represented by L markers. (**C**) Linear structure of mediterrocin with signal peptide (blue) and core sequence (black) in single-letter amino acid code. (**D**) AlphaFold predicted structure of mature mediterrocin (gray) and mediterrocin signal peptide (blue) (zoom in on core to the right).

In the active subfraction following SEC (Fig. S1B), the presence of mediterrocin was confirmed again by intact protein LC-MS/MS analysis (Fig. 2A and B). Mediterrocin was the most abundant protein in this active subfraction. The MS/MS spectrum of mediterrocin matched the expected fragmentation for the mature amino acid sequence (Fig. 2B), and only the mature bacteriocin with a cleaved signal peptide was observed by LC-MS/MS. There are no other predicted or observed post-translational modifications, as indicated by intact protein LC-MS/MS analysis (Fig. 2A and B). Thus, mature mediterrocin is an 89-residue bacteriocin (Fig. 2C).

To further confirm that mediterrocin was responsible for bioactivity, we obtained a synthetic, mature mediterrocin (Fig. S4A). The natively purified mediterrocin has identical retention time (Fig. S4B), intact mass (Fig. S4C), and fragmentation by HCD (Fig. S4C) to synthetic mediterrocin. Synthetic mediterrocin did not reproduce bioactivity at the same concentrations of protein (Fig. S4D), likely due to improper folding conditions. The AlphaFold-predicted structure of mediterrocin is a β-sheet (Fig. 2D), which is more difficult to renature due to slow folding rates (20).

### Mediterrocin is responsible for the antimicrobial activity in the active subfraction of *M. gnavus* RJX1121 CFS

**Fig 3:**
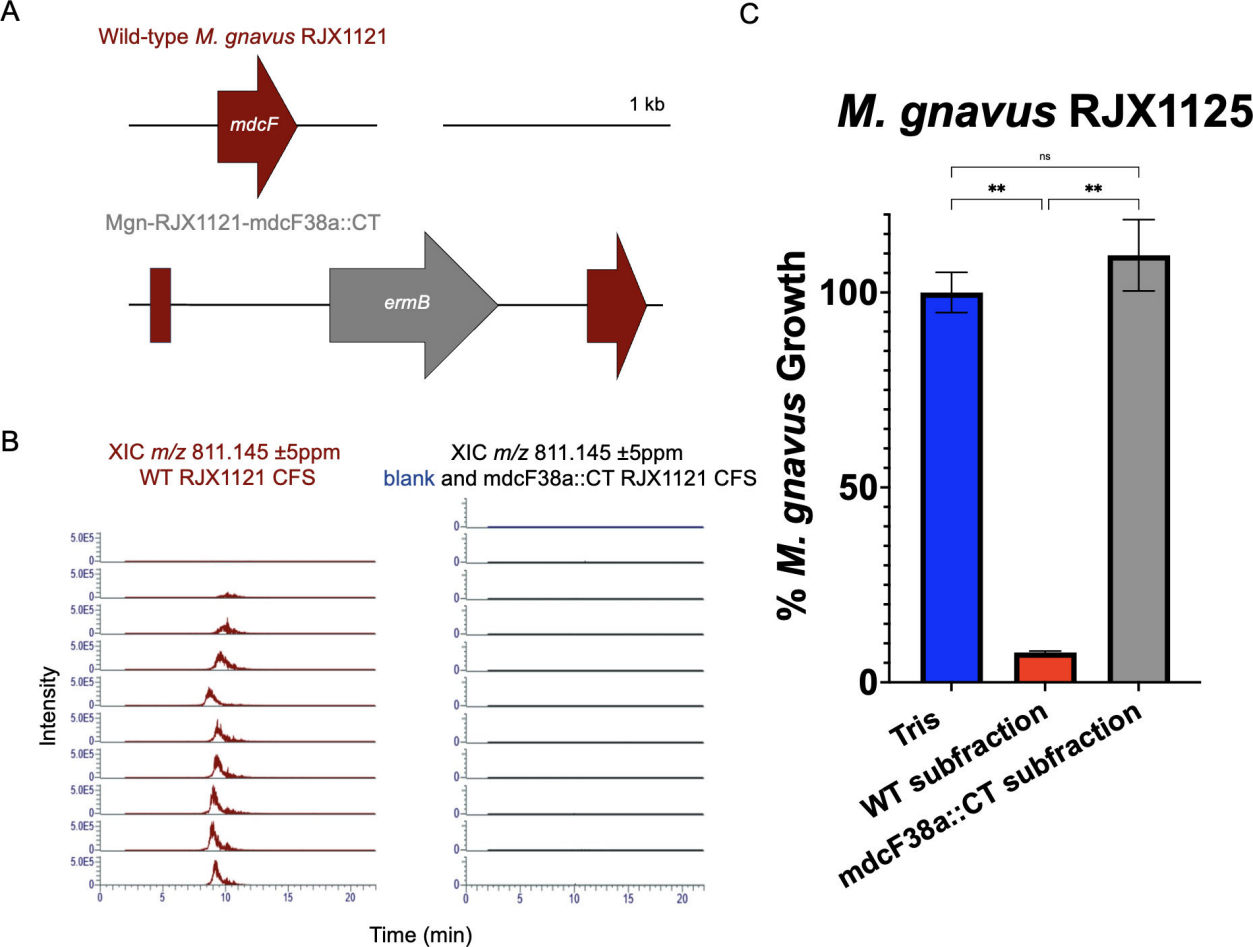
Mediterrocin disruption mutant of *M. gnavus* RJX1121 does not inhibit the growth of mediterrocin-sensitive *M. gnavus*. (**A**) Wild-type (WT) *M. gnavus* RJX1121 mediterrocin (*mdcF*) gene compared to disruption mutant (mdcF38a::CT) with *ermB* insertion. (**B**) Mediterrocin mutant strain does not produce mediterrocin as confirmed by intact protein LC-MS/MS. Extracted ion chromatograms (XICs) of *m/z* 811.145 ± 5 ppm (M + 12H)^12+^ charge state of mediterrocin in the CFS of WT RJX1121 (left, red) or mediterrocin disruption mutant RJX1121 (right, black) shown on the same scale. The column blank is shown in blue to demonstrate the baseline noise of the *m/z* 811.145 ± 5 ppm. (**C**) Percent growth of mediterrocin-sensitive *M. gnavus* RJX1125 treated with the size-exclusion subfraction from either WT or mediterrocin disruption mutant RJX1121 in 20 mM Tris buffer. Percent growth of treated to control (Tris) was calculated from the ratio of OD_600_ of treated cultures to control after 24 h of growth. Sensitivity to treatment was defined as a significant decrease in percent growth compared to 20 mM Tris control, where significance was defined as *P* < 0.05 using one-way ANOVA with Tukey’s multiple comparison testing. ** represents *P* < 0.01. Error bars represent SEM.

To confirm that antimicrobial activity from the active subfraction is due to mediterrocin, we constructed a disruption mutant of the mediterrocin gene in RJX1121 utilizing the ClosTron system (21, 22) (Fig. S5; File S1), where an erythromycin resistance gene (*ermB*) was inserted into the mediterrocin gene (Fig. 3A). We confirmed the insertion of *ermB* into the mediterrocin gene by whole-genome sequencing (File S2). We further confirmed that the mutant did not produce mediterrocin using intact protein LC-MS/MS (Fig. 3B).

The active subfraction containing mediterrocin from wild-type (WT) RJX1121 was purified in parallel with the equivalent subfraction from the mediterrocin disruption mutant (mdcF38a::CT). The WT and mutant subfractions were assayed for antimicrobial activity against mediterrocin-sensitive *M. gnavus* RJX1125. The WT subfraction containing mediterrocin, but not the mutant subfraction, was able to inhibit the growth of *M. gnavus* RJX1125 (Fig. 3C). Therefore, mediterrocin is responsible for antimicrobial activity.

### Determination of the mediterrocin BGC

**Fig 4:**
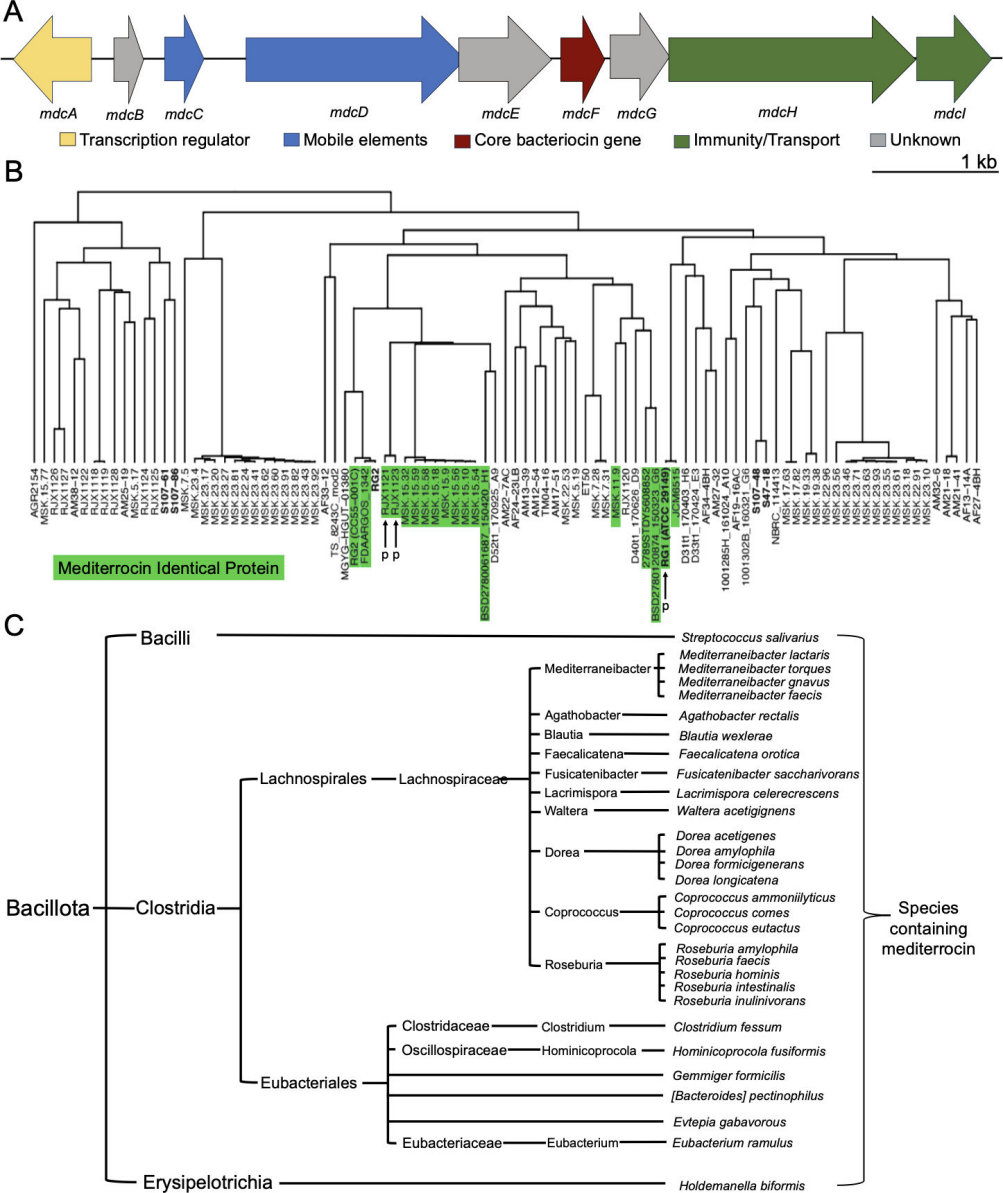
Mediterrocin gene cluster, distribution, and conservation. (**A**) Proposed mediterrocin biosynthetic gene cluster represented by open reading frames (ORFs) (CDL19_RS00960-CDL19_RS00105). Genes are named alphabetically according to the cluster order. Functional annotations are color coded according to the following scheme: yellow = transcription regulator, blue = mobile elements, red = mediterrocin gene, green = immunity to or transport of bacteriocin, and gray = unknown function. (**B**) Conservation of mediterrocin BGC within *M. gnavus* reveals a clade separation. Phylogenetic tree of *M. gnavus* strains adapted from Azzouz et al. (24) with strains that have a conserved mediterrocin gene labeled in green and producer strains used in this study (RJX1121, RJX1123, and ATCC29149) is marked with “p.” (**C**) Taxonomic tree of species in which at least one strain contains an identical protein to mediterrocin showing the distribution of the bacteriocin within Bacillota is most concentrated within Clostridia, and more specifically within Lachnospiraceae.

The mediterrocin BGC was determined through analysis of the nucleotide sequence surrounding the core mediterrocin gene (Fig. 4A). In summary, bacterial strains also possessing a mediterrocin gene were compared to find the boundaries of similarity. The genes within this region were assigned as the putative BGC. Each of the annotated open reading frames within the cluster was assigned functions if possible, using BLAST, SignalP6.0, and DeepTMHMM. The mediterrocin BGC contains a transcription regulator, mobile elements, a probable immunity protein, an ATP-binding cassette protein, and unassigned proteins including one likely secreted protein (Fig. 4A and Fig. S3A and B). Lcn972 family bacteriocin gene clusters often contain a seven-transmembrane DUF1430-containing protein, which likely confers immunity (23). Consistent with its homology to lcn972, the mediterrocin BGC contains a protein (*mdcH*) with the same DUF1430 and predicted seven-transmembrane topology (Fig. 4A and Fig. S3B). The mediterrocin gene cluster encodes an ABC-domain containing protein (*mdcI*). While ABC-domain-containing proteins are often involved in the export of mature bacteriocins, mediterrocin has an N-terminal signal peptide that is well predicted to be Sec-dependent (Fig. S3B), rather than to be dependent on a specialized transporter.

Mediterrocin and its BGC are widely distributed in Bacillota [Firmicutes], including the *M. gnavus* type strain ATCC29149. The mediterrocin gene in *M. gnavus* RJX1121 is conserved in four other *M. gnavus* strains: ATCC29149, CC55_001C, and RJX1123 with 100% sequence identity. LC-MS/MS comparison of *M. gnavus* RJX1121, RJX1123, and ATCC29149 CFSs confirmed that RJX1123 and ATCC249149 also produce mediterrocin (Fig. S6A). The expression level of mediterrocin is variable for all producer strains (Fig. S6A). The concentration of mediterrocin in RJX1121 CFS over 72 h of growth was variable (Fig. S6B). In some replicates, mediterrocin was expressed in the early stationary phase, but it was expressed in late stationary phase in others. Additionally, *Mediterraneibacter lactaris* ATCC29176, [*Bacteroides*] *pectinophilus* ATCC43243 (reclassified as Eubacteriales), *Roseburia intestinalis* NB2A 31_NB, and *Agathobacter rectalis* DSM17629 contain the mediterrocin gene.

To investigate the distribution of mediterrocin within *M. gnavus*, a phylogenetic tree constructed for *M. gnavus* (24) was annotated for strains that contain a protein sequence identical to mediterrocin (Fig. 4B). This phylogenetic tree contains clonal strains, specifically the series MSK.XX.XX that were isolated from the same subject under the BioProject ID PRJNA596270. *M. gnavus* strains which contain a mediterrocin gene clade together (Fig. 4B). Identical proteins to mediterrocin are distributed among Bacillota. Many species in which one strain contains an identical protein to mediterrocin are Clostridia which belong to either Lachnospirales or Eubacteriales (Fig. 4C). Within Lachnospirales, mediterrocin is only distributed in Lachnospiraceae, while it is distributed in multiple families within Eubacteriales.

We then annotated proteins highly similar to mediterrocin using NCBI’s BLAST. We included proteins with 100% coverage and greater than 80% sequence identity to the mediterrocin core. We allowed for any number of mutations in the signal peptide, which should not affect the activity of mature mediterrocin. We discovered many mutations in the signal peptide sequence, but the core of mediterrocin is very highly conserved (Fig. S7A). We discovered six mutation positions in the mediterrocin core, which are all predicted to be in the loops between β strands (Fig. S7A). The mediterrocin we characterize in this study is the most widely distributed sequence (Fig. S7A). Highly similar proteins to mediterrocin are also distributed among Clostridia, but not as widely distributed as mediterrocin itself (Fig. S7B).

### Mediterrocin inhibitory effects against gut commensals and pathogens

**Fig 5:**
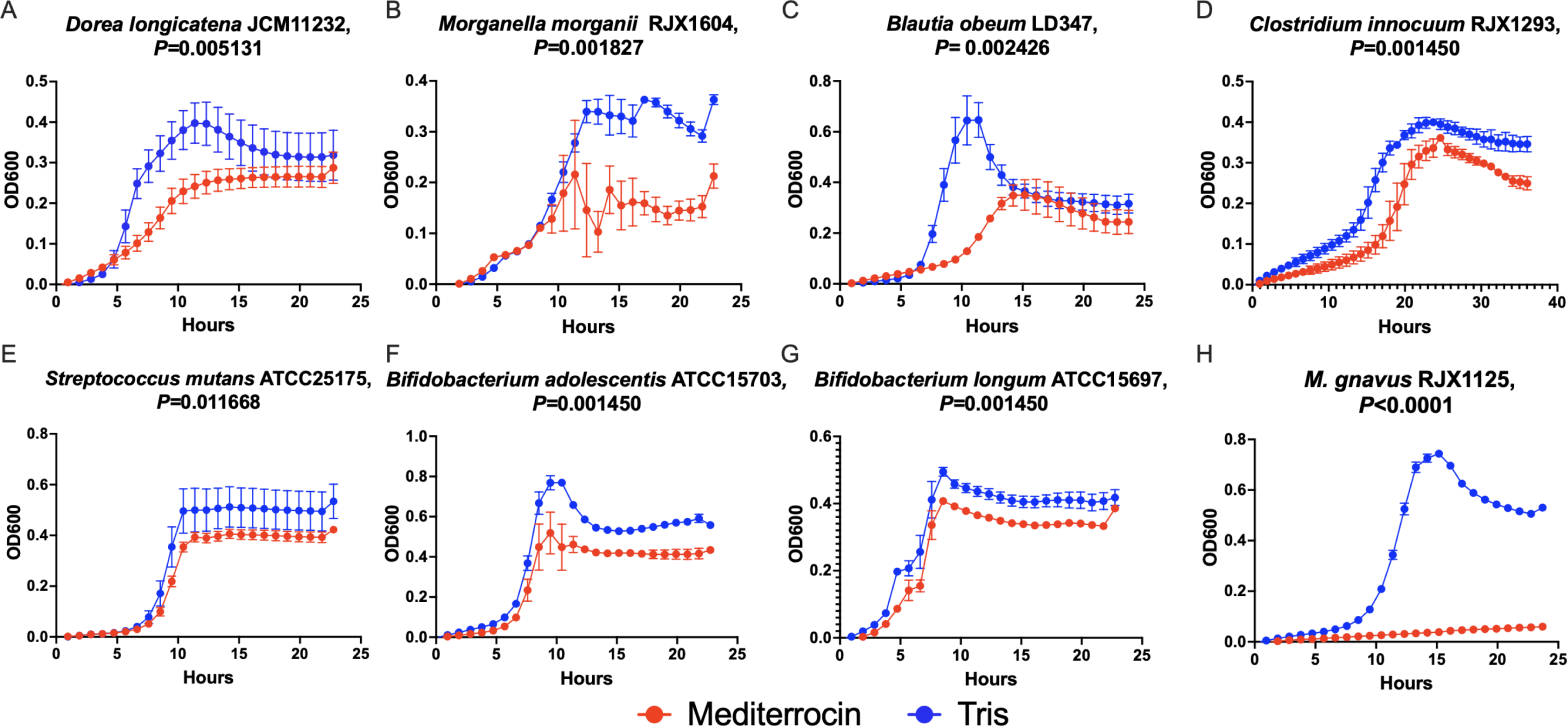
Mediterrocin displays broad-spectrum activity against members of the microbiota. Growth curves of mediterrocin-sensitive strains in a screen of 21 human microbiome bacterial strains treated with either 0.75 µg/mL mediterrocin or 20 mM Tris buffer. Sensitive strains were determined from a significant decrease in AUC of mediterrocin treatment compared to control, where significance was defined as FDR-corrected cutoff of *P* < 0.05. The strains sensitive to mediterrocin are (**A**) *Dorea longicatena* JCM11232*,* (**B**) *Morganella morganii* RJX1604, (**C**) *Blautia obeum* LD347, (**D**) *Clostridium innocuum* RJX1293, (**E**) *Streptococcus mutans* ATCC25175, (**F**) *Bifidobacterium adolescentis* ATCC15703, and (**G**) *Bifidobacterium longum* ATCC15697. (**H**) The growth curve of mediterrocin-sensitive *M. gnavus* RJX1125 is shown for comparison to non-*M*. *gnavus* sensitive strains. Error bars represent SEM.

We expanded the spectrum of activity testing of mediterrocin to a panel of 21 human microbiome strains beyond *M. gnavus*. Mediterrocin (0.75 µg/mL; 77 nM assuming 100% mediterrocin) has a broad spectrum of activity against many gram-positive species and one gram-negative. The sensitive strains include *Dorea longicatena* JCM11232*, Blautia obeum* LD347, *M. morganii* RJX1604*, Clostridium innocuum* RJX1293, *Streptococcus mutans* ATCC25175*, Bifidobacterium adolescentis*, and *Bifidobacterium longum* ATCC15697 (Fig. 5). When treated with mediterrocin, these strains had significantly reduced AUCs of their growth curves compared to untreated controls (Fig. 5). The most sensitive species outside of *M. gnavus* is *B. obeum* LD437 (Fig. 5C). *D. longicatena* and *B. obeum* are closely related Lachnospiraceae to *M. gnavus*; however, *M. morganii*, *S. mutans*, and *Bifidobacterium* sp. are more distantly related. Although mediterrocin affects the growth of these species outside *M. gnavus*, no species tested in these conditions was as sensitive as *M. gnavus* (Fig. 5). Strains insensitive to mediterrocin did not display any significant changes in growth relative to controls (Fig. S8). Screening against a wider panel of gut microbes was limited to low and variable yields from *M. gnavus* RJX1121.

### Mediterrocin is prevalent and expressed in the human gut

To investigate the relevance of mediterrocin to host health, we used clinical metaproteomics and metagenomics data sets. We used MetaQuery (25) to determine the abundance and prevalence of the mediterrocin gene in human gut metagenomes. Mediterrocin is present in 80.18% of publicly available human gut metagenome data sets (Fig. S9).

We mined publicly available microbiome metaproteomics data sets generated from human feces and GI tract sampling for annotations of mediterrocin tryptic peptides. This analysis was aided by the MetaPep spectral library (26), which mined 15 metaproteomics data sets for bacterial proteins from the human gut microbiome. MetaPep re-analyzed raw data from these cohorts to identify bacterial proteins by PSM using MetaLab MAG and pFind against the Unified Human Gastrointestinal Genome database (27). Tryptic peptides of mediterrocin were identified by MetaPep in four cohorts (PRIDE accessions: PXD007819, PXD008675, PXD011515, and PXD008870) (Table S4 to S7). These cohorts are a pediatric IBD and non-IBD cohort (28), an adult and pediatric IBD and non-IBD cohort (HMP2) (29), a type 1 diabetes (T1D) and non-T1D cohort (30), and a cohort of leukemia patients colonized with multidrug-resistant Enterobacteriaceae (MRE) (31). The cohort descriptions and number of subjects in each cohort that had annotated mediterrocin tryptic peptides are summarized in Table 2. Mediterrocin tryptic peptides were annotated by MetaPep in 15 subjects (Table S4 to S7; Table 2). Overall, the peptide fragments had 94% coverage and 100% identity to mediterrocin (Table S8). For a single subject, the greatest mediterrocin coverage was 85% and 100% identity (Table S8). Taken together, these findings demonstrate that the mediterrocin gene is prevalent and expressed in the human gut.

**TABLE 2:** Prevalence of mediterrocin tryptic peptides identified by MetaPep in metaproteomics from human feces and GI tract sampling in four cohorts^*a*^

Peptide fragment of mediterrocin identified	Cohort			
	Pediatric IBD 71 subjects (CD = 25, UC = 22, non-IBD = 24)	HMP2 132 subjects (CD = 67, UC = 38, non-IBD = 27)	T1D 101 subjects (healthy = 18, new onset = 32, seroneg = 29, seropos = 17)	Leukemia and MRE colonization 56 subjects, longitudinal study
**FSVTNGNK** TFDKKWELSTTSDGGK GVLTYGYNTLAINEDYAHAYHSTK DHYAYVKNGNGSFSGSNVGK GNWSK IEVAHKGNSITYGNSY	0/71	2/132	0/101	0/56
FSVTNGNK **TFDKKWELSTTSDGGK** GVLTYGYNTLAINEDYAHAYHSTK DHYAYVKNGNGSFSGSNVGK GNWSK IEVAHKGNSITYGNSY	1/71	0/132	0/101	0/56
FSVTNGNK TFDK **KWELSTTSDGGK** GVLTYGYNTLAINEDYAHAYHSTK DHYAYVKNGNGSFSGSNVGK GNWSK IEVAHKGNSITYGNSY	1/71	1/132	1/101	0/56
FSVTNGNK TFDKK **WELSTTSDGGK** GVLTYGYNTLAINEDYAHAYHSTK DHYAYVKNGNGSFSGSNVGK GNWSK IEVAHKGNSITYGNSY	1/71	3/132	3/101	0/56
FSVTNGNK TFDKKWELSTTSDGGK **GVLTYGYNTLAINEDYAHAYHSTK** DHYAYVKNGNGSFSGSNVGK GNWSK IEVAHKGNSITYGNSY	1/71	2/132	5/101	0/56
FSVTNGNK TFDKKWELSTTSDGGK GVLTYGYNTL **AINEDYAHAYHSTK** DHYAYVKNGNGSFSGSNVGK GNWSK IEVAHKGNSITYGNSY	0/71	0/132	6/101	0/56
FSVTNGNK TFDKKWELSTTSDGGK GVLTYGYNTLAI **NEDYAHAYHSTK** DHYAYVKNGNGSFSGSNVGK GNWSK IEVAHKGNSITYGNSY	0/71	0/132	0/101	0/56
FSVTNGNK TFDKKWELSTTSDGGK GVLTYGYNTLAINEDYAHAYHSTK **DHYAYVKNGNGSFSGSNVGK** GNWSK IEVAHKGNSITYGNSY	1/71	0/132	3/101	0/56
FSVTNGNK TFDKKWELSTTSDGGK GVLTYGYNTLAINEDYAHAYHSTK DHYAYVK **NGNGSFSGSNVGK** GNWSK IEVAHKGNSITYGNSY	1/71	6/132	0/101	1/56
FSVTNGNK TFDKKWELSTTSDGGK GVLTYGYNTLAINEDYAHAYHSTK DHYAYVKNGNGSFSGSNVGK GNWSK **IEVAHKGNSITYGNS** Y	1/71	0/132	0/101	0/56
FSVTNGNK TFDKKWELSTTSDGGK GVLTYGYNTLAINEDYAHAYHSTK DHYAYVKNGNGSFSGSNVGK GNWSK **IEVAHKGNSITYGNSY**	1/71	0/132	0/101	0/56
Total subjects with mediterrocin	1/71	7/132	6/101	1/56

^*a*^ Full sequence of mediterrocin is shown, with corresponding tryptic peptide in bold. CD, Crohn’s disease; UC, ulcerative colitis.

## DISCUSSION

Using an inter-strain competition screen of *M. gnavus* strains, we identified mediterrocin, a broad-spectrum bacteriocin, in the CFS of *M. gnavus* RJX1121. The protein was identified via LC-MS/MS intact protein analysis. We confirmed the gene that encodes mediterrocin is responsible for antimicrobial activity by constructing a disruption mutant. Purified mediterrocin significantly decreases the growth curve AUC of 4 out of 10 strains of *M. gnavus* tested: RJX1119, RJX1125, RJX1128, and ATCC35913. RJX1128 and ATCC35913 are the most sensitive strains with an MIC of roughly 125 nM. As expected, strains that produce mediterrocin are not sensitive to it because they contain a putative immunity protein.

*M. gnavus* is phylogenetically separated into so-called health-associated and IBD-associated clades (15, 32). Many IBD-associated strains elicit pro-inflammatory cytokine secretion in innate immune cells, while health-associated strains produce a tolerogenic capsule that blocks immune detection (33). The mediterrocin gene is only found in strains within the healthy-associated clade. Multiple pro-inflammatory strains belonging to the disease-associated clades are sensitive to mediterrocin, while tolerogenic strains are resistant to mediterrocin. However, ATCC35913, a healthy-associated isolate from the 1970s, is highly sensitive to mediterrocin, and RJX1122 and RJX1124, disease-associated isolates, are resistant to mediterrocin. *M. gnavus* strain CC55_001C, which produces an immunogenic lipoglycan implicated in lupus (16), also has the mediterrocin gene. Beyond mediterrocin, *M. gnavus* produces other molecules that affect both host immune responses and other microbes. These include inflammatory cell wall polysaccharides (34–36), tolerogenic capsules (34), immunogenic lipoglycans (16), and bacteriocins (17, 18, 37). These factors likely interact and contribute to how a given strain will behave in the gut of an individual and highlight that this species should not be treated as a monolith. The relevance of mediterrocin in health and whether it could serve to prevent pro-inflammatory *M. gnavus* from colonizing the gut while not disturbing tolerogenic *M. gnavus* strains will be the subject of future studies.

During spectrum of activity screening, mediterrocin inhibited commensal and pathogenic bacteria beyond *M. gnavus*. Mediterrocin is most potent against other *M. gnavus* and closely related Lachnospiraceae, but inhibits the growth of a broad spectrum of species. The broad spectrum of activity of mediterrocin is unsurprising considering the wide distribution of the mediterrocin gene. Bacteriocins with broad-spectrum activity are less common, especially with activity against both gram-positive and gram-negative bacteria (7). Previously characterized bacteriocins from *M. gnavus* have also demonstrated broad-spectrum activity (17, 38), for example, ruminococcin A from *M. gnavus* E1 inhibits *M. gnavus* ATCC29149, *B. longum*, *B. adolescentis*, *B. obeum*, and multiple gram-negative species (17).

At the concentration tested (0.75 µg/mL, 77 nM assuming 100% purity), mediterrocin most potently inhibited *M. gnavus* when compared to other microbiome species. Further testing at higher concentrations and against a greater variety of bacteria will be required to determine the most sensitive species and strains. MIC determination of mediterrocin against each of these sensitive strains will clarify a potential ecological role of mediterrocin from trends in activity. From our characterization, mediterrocin is most effective at preventing the growth of particular *M. gnavus* strains. Due to the reduced GC content (34.5%) of the mediterrocin gene compared to the genome sequence (42.9%), the mobile elements and integrase surrounding the mediterrocin gene, and the distribution of mediterrocin in Bacillota, this bacteriocin was likely acquired by a horizontal transfer event. Whether mediterrocin functions as an *M. gnavus*-specific antimicrobial produced by a variety of gut commensals is a topic of continued study. Further study of the ecological role of mediterrocin in the human gut may explain why mediterrocin is distributed among Bacillota, particularly the Lachnospiraceae, and why only some strains of *M. gnavus* are highly sensitive.

We observed variable expression and yield of mediterrocin in culture broth. Low mediterrocin yields in large-scale culture of *M. gnavus* RJX1121 limited the testable concentration range in assays and our spectrum of activity screening. Transcriptomics analysis of *M. gnavus* ATCC29149 revealed that mediterrocin expression is influenced by the presence of mucin, fucose, and galactose in growth medium (38). The variability of expression of mediterrocin and its regulation will be subjects for further study and may clarify the ecological role of mediterrocin.

The mechanism of action of mediterrocin is a topic of continued study. Based on the structural similarity of mediterrocin to lcn972, it is possible that mediterrocin may also target the cell wall. Lcn972 interacts with lipid II to inhibit cell wall synthesis during septum formation (39, 40). Further study of mediterrocin will investigate potential interactions with lipid II or another cell wall synthesis target. Unlike mediterrocin, lcn972 is a narrow-spectrum bacteriocin which only inhibits *Lactococcus lactis* species (41). The structure-activity relationship between mediterrocin and *M. gnavus* sensitivity should also be investigated. The mechanism of action may be revealed by studying strains that lack the mediterrocin immunity protein yet are resistant to mediterrocin in comparison to mediterrocin-sensitive strains.

The ~80% prevalence of mediterrocin in human gut metagenomes determined using MetaQuery is likely due to the wide distribution of mediterrocin. Similarly, the mediterrocin peptide fragments identified in metaproteomics data sets are not necessarily produced by *M. gnavus* due to the high homology of mediterrocin in other species. Regardless of which organism produces mediterrocin, the fact that it is expressed in people warrants continued investigation. Moreover, the prevalence of mediterrocin expression in people, as shown in Table 2 is a conservative lower bound, as MetaPep uses peptide spectral matching to tryptic peptides. More sensitive detection methods for mediterrocin, either with an antibody or targeted MS-based feature detection, should be carried out in the future to fully determine its expression in people.

Future investigation of mediterrocin *in vivo* could aid studies of the differential abundance of specific strains of *M. gnavus* observed in dysbiotic gut microbiomes in diseases including IBD and lupus. Perhaps the loss of the tolerogenic mediterrocin producer strains allows for the invasion of inflammatory strains, which are usually kept out in part by mediterrocin. Mediterrocin may function as a niche protective bacteriocin against some *M. gnavus* from the disease-associated clades. This role of bacteriocins as a strategy for colonization resistance by commensals in the inflamed gut has been described for Enterobacteriaceae-produced microcins (11). Understanding microbial competition in the gut through bacteriocin production will continue to inform how microbial gut ecology contributes to disease and allow for strain rather than species-targeted therapeutics.

## MATERIALS AND METHODS

### Bacterial strains and growth

Strains used in this study (Table 3) were procured as glycerol stocks from ATCC and collaborators. All strains were grown anaerobically (∼5% H_2_, 85% N_2_, and 10% CO_2_) at 37°C in defined medium (DM) (42) or Bacto reinforced clostridial medium (RCM).

**TABLE 3:** Strains used in this study^*a*^

Species	Strain	Original isolation source
*Mediterraneibacter gnavus*	ATCC29149	Healthy adult feces
*Mediterraneibacter gnavus*	ATCC35913	Healthy adult feces
*Mediterraneibacter gnavus*	RJX1119	Infant treated with antibiotics (43)
*Mediterraneibacter gnavus*	RJX1120-RJX1127	IBD patient biopsy (15)
*Mediterraneibacter gnavus*	RJX1128	Feces of IBD patient (15)
*Dorea longicatena*	JCM11232	
*Blautia obeum*	LD347	
*Clostridium nexile*	RJX1984	
*Mediterraneibacter torques*	L2-14	
*Enterococcus faecalis*	920_EFLS	
*Enterococcus faecalis*	DIAB237	
*Lactococcus lactis*	DIAB158	
*Streptococcus mutans*	ATCC25175	
*Enterococcus faecium*	DIAB134	
*Enterococcus avium*	DIAB219	
*Clostridium innocuum*	RJX1293	
*Morganella morganii*	RJX1604	
*Citrobacter freundii*	ATCC8090	
*Eggerthella lenta*	DIAB165	
*Bifidobacterium adolescentis*	ATCC15703	
*Bifidobacterium longum*	ATCC15697	
*Bacteroides thetaiotaomicron*	sp. 1_1_16	
*Bacteroides thetaiotaomicron*	VPI-5482	
*Parabacteroides distasonis*	ATCC8053	
*Coprococcus comes*	LD003	
*Roseburia inulinivorans*	DSM16841	
*Roseburia intestinalis*	L1-82	

^*a*^ The empty cells indicate that origin information was not specified for species other than *M. gnavus*, as the study focused on this organism and included multiple isolates of it.

### Purification of mediterrocin from *M. gnavus* RJX1121 CFS

Starter cultures of *M. gnavus* RJX1121 were grown in DM from single colonies on solid DM. Large-scale cultures were inoculated from the starter culture in a 1:1,000 ratio in DM and grown for 3 days. CFS was generated through centrifugation and filtration using 0.2 µm PES vacuum filters. CFS was concentrated 10× and desalted by precipitation with four volumes of acetone. Protein pellets were resuspended in 20 mM Tris, pH 7.5. The concentrated CFS was purified using a Cytiva HiTrap Q FF 5 mL anion exchange column on an ÄKTA go FPLC with a gradient of 0–100% 1 M NaCl, where mediterrocin eluted at 0.1 mM NaCl. The SAX fraction was precipitated with four volumes of acetone. The pellet was resuspended in 20 mM Tris. Mediterrocin was further purified with a Cytiva Superdex 75 Increase column with a flow rate of 0.6 mL/min 20 mM Tris.

Fractions generated during purification efforts were tested for the ability to inhibit the growth of *M. gnavus* RJX1124 or RJX1125. Starter cultures of *M. gnavus* were prepared as described above and diluted with DM to OD_600_ = 0.6 using an OD meter and further diluted with DM in a 1:1,000 ratio. 20 µL of fractions or Tris buffer was combined with 80 µL of 1:1,000 cultures and incubated overnight. OD_600_ was monitored using a Cerillo Stratus plate reader.

### Mediterrocin spectrum of activity assays

*M. gnavus* cultures were prepared as described above. 10 µL of *M. gnavus* RJX1121 CFS, concentrated CFS, purified mediterrocin (6.25, 12.5, and 25 µg/mL) in 20 mM Tris, 20 mM Tris, or additional DM were combined with 90 µL of *M. gnavus* 1:1,000 cultures and incubated overnight. OD_600_ was monitored using a Cerillo Stratus. DM treatment served as a control for CFS treatment, and Tris treatment served as a control for concentrated CFS and purified mediterrocin treatment. Statistical analysis was performed with GraphPad Prism to calculate AUCs of treatment and control growth curves and then perform Student’s *t*-tests where significance was defined as *P* < 0.05. Treatment sensitivity of *M. gnavus* was defined as a significant decrease in AUC compared to control. As an alternative definition, percent growth of treated cultures at the time of maximum OD_600_ of untreated controls was calculated. Mediterrocin sensitivity of *M. gnavus* was determined by one-way ANOVA with Tukey’s multiple comparison testing of *M. gnavus* percent growth using GraphPad Prism, where significance was defined as *P* < 0.05.

A panel of gut commensals was screened for inhibition by mediterrocin by inoculating starter cultures in DM or RCM from glycerol stocks. 1 µL of the starter cultures was passaged in 1 mL media. Purified mediterrocin (7.5 µL; 7.5 µg/mL) in 20 mM Tris or buffer was combined with 67.5 µL of inoculated cultures and incubated. OD_600_ was monitored using a Cerillo Stratus. Statistical analysis was performed with GraphPad Prism to calculate AUCs of treatment and control growth curves and then perform multiple FDR corrected *t*-tests using the Benjamini-Hochberg method with a 5% FDR, and significance was defined as FDR adjusted *P* < 0.05. All experiments were performed in triplicate.

### LC-MS/MS characterization and quantitation of mediterrocin

Sample injections of 5 µL were used for CFS and 10 µL for purified mediterrocin. Proteins were separated for MS analysis on a ProSwift RP-4H Monolithic Capillary 100 µm × 25 cm column using a gradient of 95% water (0.1% formic acid) to 100% acetonitrile (0.1% formic acid) over 15 mins and analyzed by LC-MS/MS (Vanquish HPLC and Orbitrap Exploris 120, Thermo Fisher Scientific). Chromatographic and MS data were acquired with Xcalibur (Thermo Fisher Scientific). MS data were collected in the range of *m/z* 500–2,000. MS/MS data were collected with a normalized fragmentation energy of HCD 30 V. Purified mediterrocin was retention time and MS/MS matched to synthetic mediterrocin. Protein sequence analysis was performed using the Xcalibur protein Xtract tool to generate intact monoisotopic masses for ProSight Lite (44) and manual sequence annotation. Comparisons of mediterrocin expression between *M. gnavus* strains at 24 h of growth and of RJX1121 time points were performed with a targeted scan range of *m/z* 600–1,400. Freestyle was used to integrate peak areas of Extracted Ion Chromatograms (XICs) for the *m/z* 811.145 ± 5 ppm. Mediterrocin expression comparisons between strains were performed with *n* = 3, and RJX1121 mediterrocin expression over time was performed with *n* = 6.

### ClosTron mutagenesis

*M. gnavus* mutants were generated as described in the Clostron-mediated engineering of Clostridium (21) to insert an erythromycin resistance cassette to disrupt the mediterrocin gene and confirmed with whole-genome sequencing and intact protein LC-MS/MS. An extended description can be found in the Supplemental methods.

In parallel, the active SEC subfraction containing mediterrocin was purified from 100 mL cultures of the WT and mediterrocin disruption mutant of *M. gnavus* RJX1121 according to the purification scheme described above. The bioactivity of the subfractions was assayed against *M. gnavus* RJX1125 using 10 µL of the subfractions or 20 mM Tris and 90 µL of a 1:1,000 diluted starter culture of the indicator strain. Following a 24-h incubation, the OD_600_ was measured using a BioTek plate reader to calculate the percent growth of cultures treated with subfractions or Tris. Statistical analysis of a one-way ANOVA with Tukey’s mutliple comparison testing was performed with GraphPad Prism, where significance was defined as *P* < 0.05.

### Annotation of the mediterrocin BGC

The boundaries of the putative BGC were determined through comparison of sequence homology in strains containing genes encoding mediterrocin. SignalP6.0 (45) was used to predict the signal peptide of mediterrocin from the amino acid sequence. DeepTMHMM (46) was used to predict the transmembrane topology and likelihood of secretion of proteins in the mediterrocin BGC. Sequence alignment of mediterrocin to lactococcin 972 was made using ESPript (47). The core sequence of the AlphaFold predicted structure of mediterrocin and lactococcin 972 (PDB ID: 2LGN) was aligned using TM-align (48) and visualized in ChimeraX. The *M. gnavus* phylogenetic tree was adapted from Azzouz et al. (24). *M. gnavus* strains that contain an identical protein sequence to mediterrocin annotated by BLAST were highlighted in green.

## Data Availability

Intact protein analysis LC-MS/MS data are available through MassIVE data set MSV000095709 (doi:10.25345/C5BK17182).

## SUPPLEMENTAL MATERIAL

File S1 mbio.01523-25-s0001.txt Plasmid sequence of pmH-Mgn-mdcF used to generate mediterrocin disruption mutant.

File S2 mbio.01523-25-s0002.txt Whole genome assembly of mediterrocin disruption mutant *M. gnavus* RJX1121 mdcF38a::CT.

Supplemental Material mbio.01523-25-s0003.docx Supplemental methods and figures, Table S1, and captions for Files S1 and S2 and Tables S2 to S8.

Supplemental Tables mbio.01523-25-s0004.xlsx Tables S2-S8.

ASM does not own the copyrights to Supplemental Material that may be linked to, or accessed through, an article. The authors have granted ASM a non-exclusive, world-wide license to publish the Supplemental Material files. Please contact the corresponding author directly for reuse.
