# Supplementary material for: A core human gut microbe, Mediterraneibacter gnavus, produces a broad-spectrum bacteriocin mediterrocin
Source: mBio. 2025 Jul 17;16(8):e01523-25. doi: 10.1128/mbio.01523-25 (PMC12345188; doi:10.1128/mbio.01523-25)
Supplement: Supplemental Material — Supplemental methods and figures, Table S1, and captions for Files S1 and S2 and Tables S2 to S8. [file mbio.01523-25-s0003.docx]

**Supplemental Materials for**

A core human gut microbe, *Mediterraneibacter gnavus*, produces a broad-spectrum bacteriocin mediterrocin

**Extended Methods**

***M. gnavus* competition screen assay.** Starter cultures of *M. gnavus* strains were grown anaerobically at 37°C in DM from an agar plate of defined medium (DM). The cultures were then diluted with DM in a 1:1000 ratio. 200µL of the 1:1000 cultures were grown in a 96-well plate for 3 days at 37°C. On day 2, a new set of starter cultures of *M. gnavus* RJX1119-28, ATCC29149, and ATCC35913 were grown overnight at 37°C in DM from an agar plate of DM. Cell-free supernatant (CFS) was generated through centrifugation of the 96-well plate to remove cells and filtered using 0.2-μm PALL 96-well filter plate. The starter cultures were diluted with DM in a 1:1000 ratio. 10µL of CFS generated from each strain or additional DM were combined pairwise with 90µL of 1:1000 cultures of each strain and grown overnight at 37 °C in DM. Following 16 hour incubation, the OD600 was measured using a BioTek plate reader and used to calculate percent growth of cultures treated with *M. gnavus* CFS or media.

**Initial characterization of mediterrocin as a bacteriocin.** A starter cultures of *M. gnavus* RJX1121 were grown at 37 °C in DM from an agar plate of DM. 10 mL cultures were inoculated from the starter culture in a 1:1000 ratio in DM and grown at 37 °C for 3 days. CFS was generated through centrifugation and filtration using 0.2-μm PES vacuum filters. CFS was filtered using a 10kD molecular weight cutoff spin filter (Millipore) to generate a fraction containing proteins greater than 10kD which are retained on the filter and a fraction containing proteins less than 10kD which flow through the filter. CFS was subjected to boiling at 100 °C for 1 hour using a heating block. The boiled CFS was centrifuged to remove insoluble protein before bioactivity testing. The fractions were assayed for the ability to inhibit the growth of *M. gnavus* RJX1124. A starter culture of *M. gnavus* RJX1124 was prepared as described above and diluted with DM in a 1:1000 ratio. 10µL of fractions or additional DM were combined with 90µL of 1:1000 starter culture and grown at 37°C for 16 hours. OD600 was measured using a BioTek plate reader and used to calculate percent growth of cultures treated with fractions or DM.

**Identification of mediterrocin with trypsin digestion.** 1 µg proteins were resuspended in 15 µL of 8M urea/100 mM Tris buffer (pH 8.5). 1.5 µL of 50 mM TCEP (product no. 646547, Sigma-Aldrich, USA) was added and incubated at room temperature for 20 minutes. Then, 1.6 µL of 500 mM 2-chloroacetamide (A15238, Alfa Aesar, USA) was added and incubated for 15 minutes in the dark at room temperature. The reaction was diluted with 3 volumes of 100 mM Tris buffer pH 8.5 to reduce the urea concentration to 2M before trypsin (T1426, Sigma-Aldrich, USA) was added to a final protease:protein ratio of 1:50 (w/w) and incubated overnight at 37°C. After that, formic acid was added to a final concentration of 1% and samples were analysed by liquid chromatography-mass spectrometry (LC/MS). Peptides obtained from the previous step were loaded onto Evotips and cleaned up following the manufacturer’s instructions (Evosep Biosystems, Denmark). Samples were injected into a Thermo Q Exactive HF quadrupole-Orbitrap MS equipped with a nanospray ESI source (Thermo Fisher Scientific, USA) using an Evosep One instrument and endurance column (Evosep Biosystems). The standard preset method 30 samples per day (30 SPD) was used for the LC component of the run. The spray voltage was set at 1.9 kV. The mass spectrometer was operated in positive ionization and data dependent acquisition mode, automatically switching between MS1 and MS2 spectra. The MS1 scan range was 200-2000 m/z with a resolution of 60,000 and MS1 automatic gain control (AGC) target value of 3e6. Up to 15 peptide precursors were selected for MS2 analysis with an isolation width of 2 m/z at the resolution of 30,000 and AGC target of 1e5.The maximum injection time for both MS1 and MS2 was 100 ms. The normalized collision energy (NCE) was set at 30% for ion fragmentation by HCD. RAW files were searched with MSfragger in FragPipe v20.0 against *Mediterraneibacter gnavus* (strain CC55_001C) reference database (Uniprot UP000018690, accessed July 2023). The precursor and fragment mass tolerances were set at 20 ppm. The maximum missed cleavage was 2. Carbamidomethylation of cysteine was set as fixed modification and N-terminal acetylation and methionine oxidation were set as variable modification. The false discovery rate was set at 1% at protein level. Other parameters were used as default. The mediterrocin gene was identified through NCBI BLAST of the peptide sequence against the *M. gnavus* RJX1121 genome assembly in NCBI.

**Synthetic mediterrocin.** The mature sequence of mediterrocin was ordered to be synthesized by LifeTein using solid phase synthesis. Synthetic mediterrocin was resuspended in 20 mM Tris pH 7.5 at 5 mg/mL, then diluted with 8M urea to denature the protein. The protein was then dialyzed back to 20 mM Tris and minimal urea using a ThermoScientfic 3.5 kDa slide-a-lyzer and 3 rounds of dialysis in 20 mM Tris pH 7.5 to yield a final concentration of 0.5 mg/mL protein. A urea control was generated by adding the equivalent volumes of 20 mM Tris and 8M urea to a 3.5 kDa slide-a-lyzer and dialyzing the buffer back to 20 mM Tris. This was found to be the optimal folding conditions, but did not reproduce antimicrobial effects of the native protein. Antimicrobial activity of the dialyzed synthetic protein and urea control was evaluated using the same activity assay as described for the spectrum of activity assays against *M. gnavus* RJX1125.

**ClosTron mutagenesis.** *M. gnavus* mutants were generated as described in the Clostron-mediated engineering of Clostridium (21) to insert an erythromycin resistance cassette to disrupt the mediterrocin gene. The target site at nucleotide 38 and placed in the antisense direction was identified using the Perutka method (22). The retargeted intron was synthesized and ligated into the pMTL-Mod2 plasmid by GenScript. The plasmid was transformed into *E. coli* HB101/prk24 following the Q5 site-directed mutagenesis kit where heat shock at 42 °C for 30 seconds was followed by 5 minutes incubation on ice before the recombinant clones were selected for chloramphenicol resistance (25 μg ml^-1^). Recombinant *E. coli* was grown overnight aerobically in 5 mL Luria–Bertani broth (LB) supplemented with chloramphenicol (12.5 µg mL^-1^). 1 mL of the overnight culture was pelleted and washed with PBS. *M. gnavus* was grown concurrently overnight in 1 mL of DM. The *E. coli* cell pellet was brought in the anaerobic chamber and resuspended in 200 μL of the overnight *M. gnavus* culture. Drops from the mixture were spotted onto a non-selective Todd Hewitt (TH; Bacto) plate. Following overnight incubation at 37 °C, the colonies were scraped and washed with 1 mL PBS and plated onto TH supplemented with cycloserine (250 µg mL^-1^) and thiamphenicol (15 µg mL^-1^). After overnight incubation, individual colonies were grown in non-selective TH broth overnight. The culture was then plated on TH supplemented with cycloserine (250 µg mL^-1^) and erythromycin (2.5 µg mL^-1^) to select for *M. gnavus* cells that successfully incorporated the erythromycin cassette in the targeted gene. The insertion of *ermB* into the mediterrocin gene was confirmed with whole genome sequencing of the mutant (Plasmidsaurus).

The loss of mediterrocin production in the mutant strain was confirmed by intact protein LC-MS/MS. CFS was generated from 24 hour cultures (n=10) of the WT and mutant RJX1121 strains and analyzed by intact protein LC-MS/MS. Extracted ion chromatograms (XICs) for the *m/z* 811.145 ± 5 ppm were used to evaluate mediterrocin expression.

**Metaproteomics and metagenomics analysis of mediterrocin prevalence.** The amino acid sequence of mediterrocin was searched in the MetaPep spectral library (26) and identified in the following datasets: PXD007819, PXD008675, PXD011515, and PXD008870. These tryptic peptides were then searched in the pFind output tables for each dataset and extracted to generate tables S4-7. For each mediterrocin tryptic peptide annotation in each cohort, the protein sequence coverage, percent identity to mediterrocin, and range of pFind scores were calculated.

MetaQuery (25) was used to determine the abundance and prevalence of the mediterrocin gene in human gut metagenomics datasets using the following parameters: minimum percent identity: 98, maximum E-value: 1e-5, minimum query alignment coverage: 90, and minimum target alignment coverage: 90. This method identified no homologs of mediterrocin and the output includes only mediterrocin (WP_005608346.1).

**Supplementary Figures and Tables**


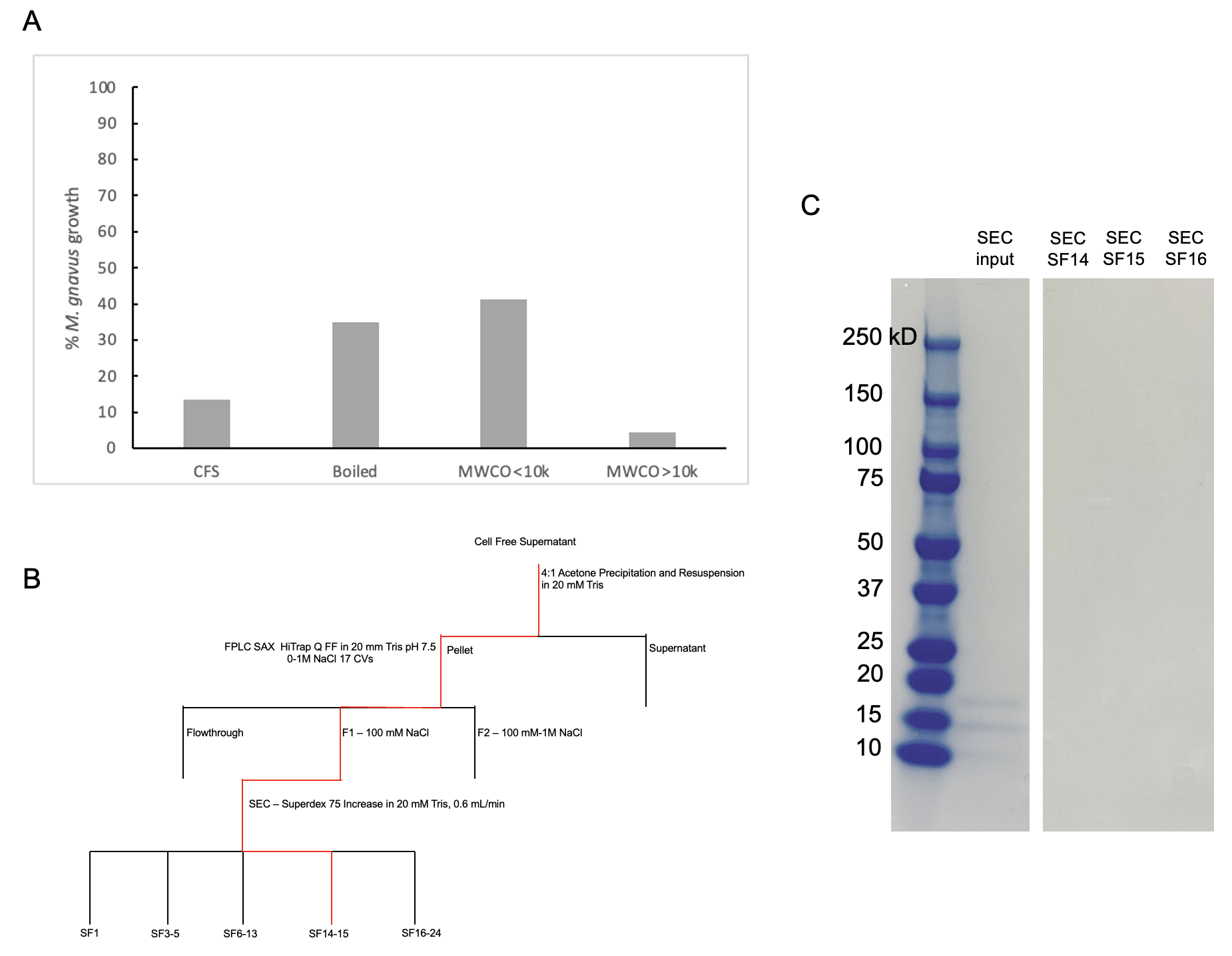


**Fig. S1. (A)** Initial characterization of mediterrocin as bacteriocin following heat treatment and filtration through molecular weight cutoff (MWCO) filters of 10kD compared to untreated *M. gnavus* RJX1121 cell-free supernatant (CFS). The indicator strain *M. gnavus* RJX1124 was treated with CFS, boiled CFS, or MWCO filtered CFS. Average percent growth (n=3) of the indicator strain was calculated from the ratio of OD600 of treated cultures to untreated culture. **(B)** Fractionation scheme of mediterrocin from *M. gnavus* RJX1121 CFS using strong anion exchange (SAX) followed by size-exclusion chromatography (SEC). **(C)** SDS-PAGE of SEC subfractions using a 4-20% polyacrylamide protein gel. Ladder of protein standards shown on the left with molecular weights. SEC fractions were generated from the input material shown in lane 1. Protein bands were visualized with Coomassie G-250 blue stain.


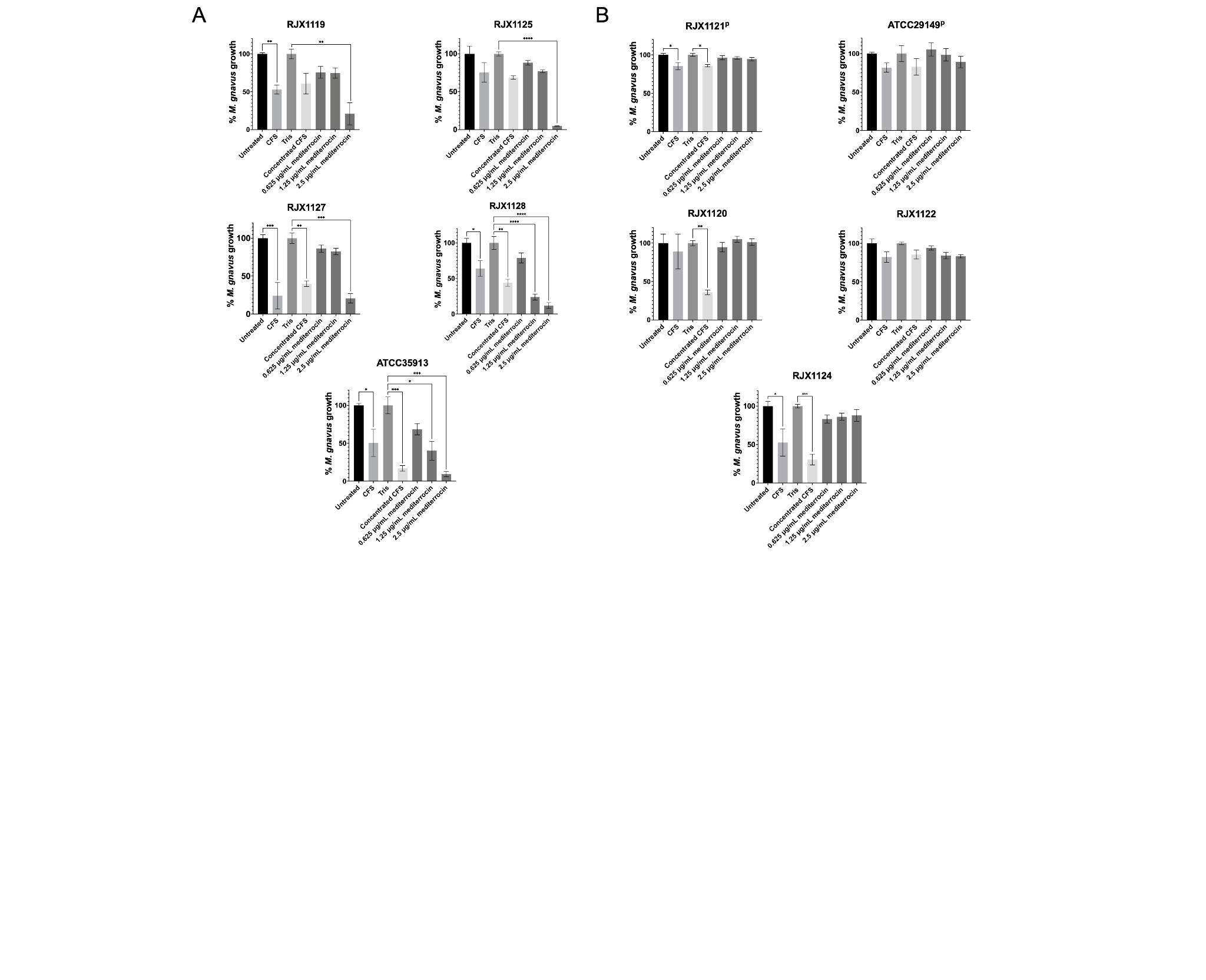


**Fig. S2.** Percent growth of *M. gnavus* strains treated with *M. gnavus* RJX1121 CFS, concentrated CFS, and a dilution series of purified mediterrocin in 20 mM Tris. Percent growth of treated to control (untreated or tris) was calculated from the ratio of OD600 of treated cultures to untreated culture at the time of maximum optical density (maxOD600) of the control. **(A)** Mediterrocin sensitive *M. gnavus* strains and **(B)** insensitive strains. Mediterrocin sensitivity of *M. gnavus* was determined by one-way ANOVA with Tukey’s multiple comparison testing of *M. gnavus* percent growth when treated with RJX1121 CFS, concentrated CFS, or purified mediterrocin compared to untreated and Tris treated controls where significance was defined as *p* < 0.05.* represents *p* < 0.05, ** represents *p* <0.01, *** represents *p* <0.001, and **** represents *p* <0.0001. Error bars represent SEM. *M. gnavus* strains RJX1121 and ATCC249149, which produce mediterrocin, are identified with a superscript p.

**
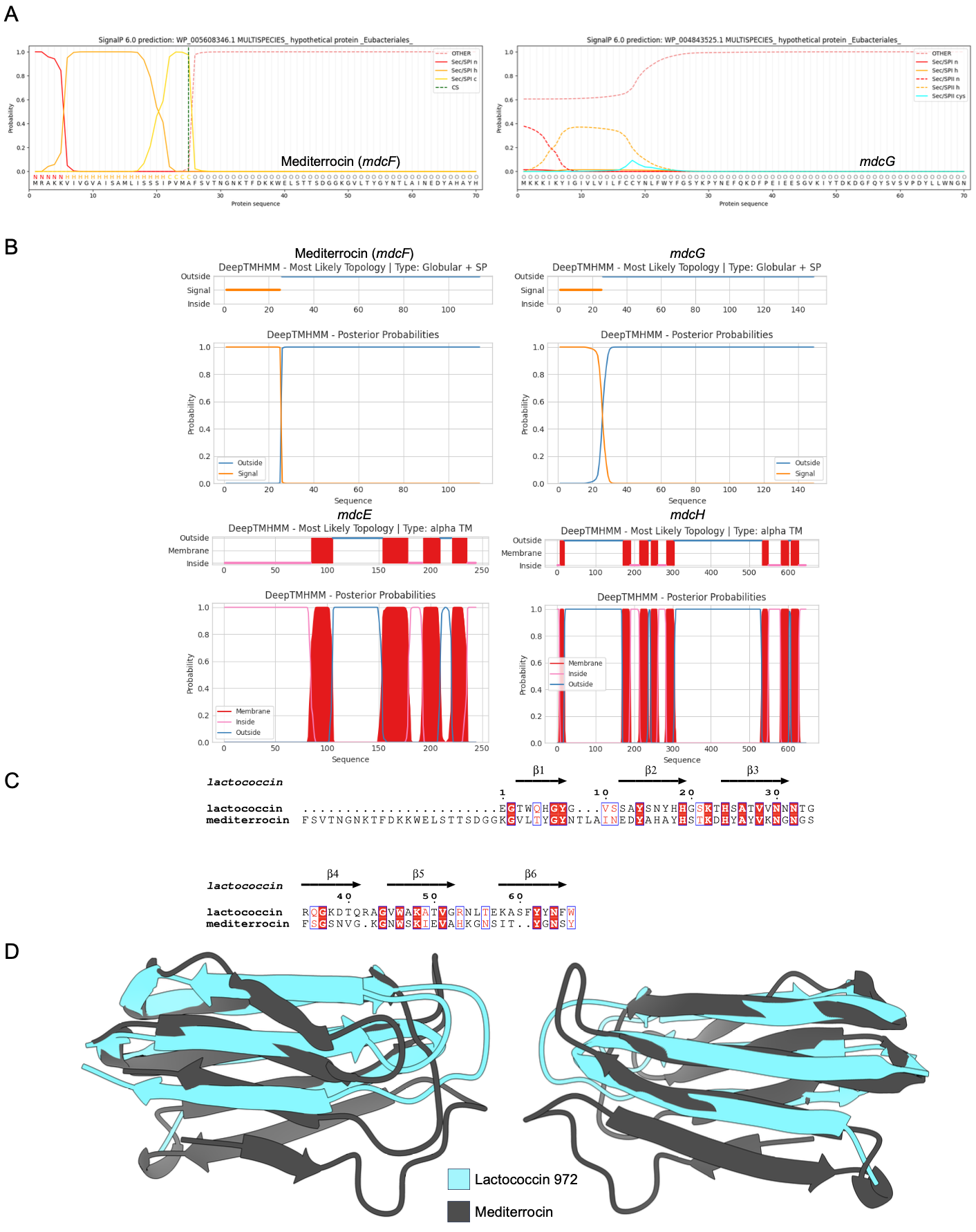
**

**Fig. S3. (A)** Signal peptide prediction of the mediterrocin gene (*mdcF*) and *mdcG* (CDL19_RS00995) by signalP6.0. **(B)** Protein topology predictions of *mdcF*, *mdcG*, *mdcE* (CDL19_RS00985), and the predicted immunity protein encoded by *mdcH* (CDL19_RS001000) by DeepTMHMM. **(C)** Sequence alignment of mediterrocin to lactococcin 972 made using ESPript shows low (18%) sequence identity. **(D)** ChimeraX visualization of the alignment of the AlphaFold predicted mediterrocin structure (gray) to lactococcin 972 (blue) (PDB ID: 2LGN) using TM-align.


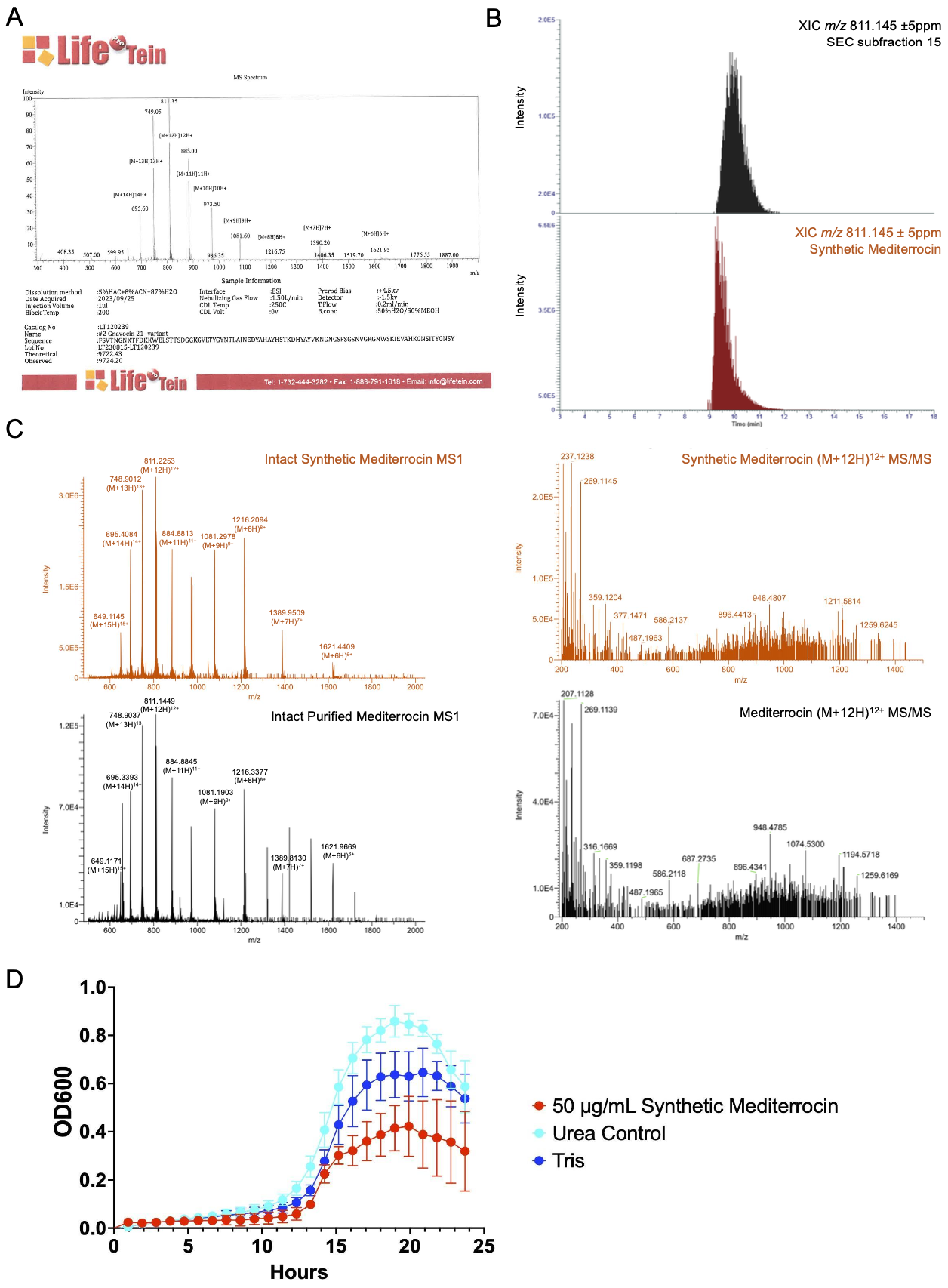


**Fig. S4. (A)** LifeTein confirmation of mediterrocin sequence by LC-MS. **(B)** Natively purified mediterrocin has identical retention time as synthetic mediterrocin. Extracted ion chromatograms (XICs) of *m/z* 811.145 ±5 ppm (M+12H)^12+^ charge state of purified mediterrocin (black trace) and mediterrocin synthetic standard (red trace). **(C)** LC-MS/MS matching of natively purified mediterrocin to synthetic mediterrocin. Intact protein analysis MS1 spectra of the synthetic mediterrocin standard and mediterrocin purified from *M. gnavus* RJX1121 cell-free supernatant. MS/MS spectra of precursor *m/z* 811.22 (M+12H)^12+^ charge state of synthetic mediterrocin and mediterrocin from *M. gnavus* RJX1121 cell-free supernatant. **(D)** Growth curves of *M. gnavus* RJX1125 treated with 50 µg/mL dialyzed synthetic mediterrocin (red), dialyzed urea control (cyan), or 20 mM Tris (blue). Error bars represent SEM.

**
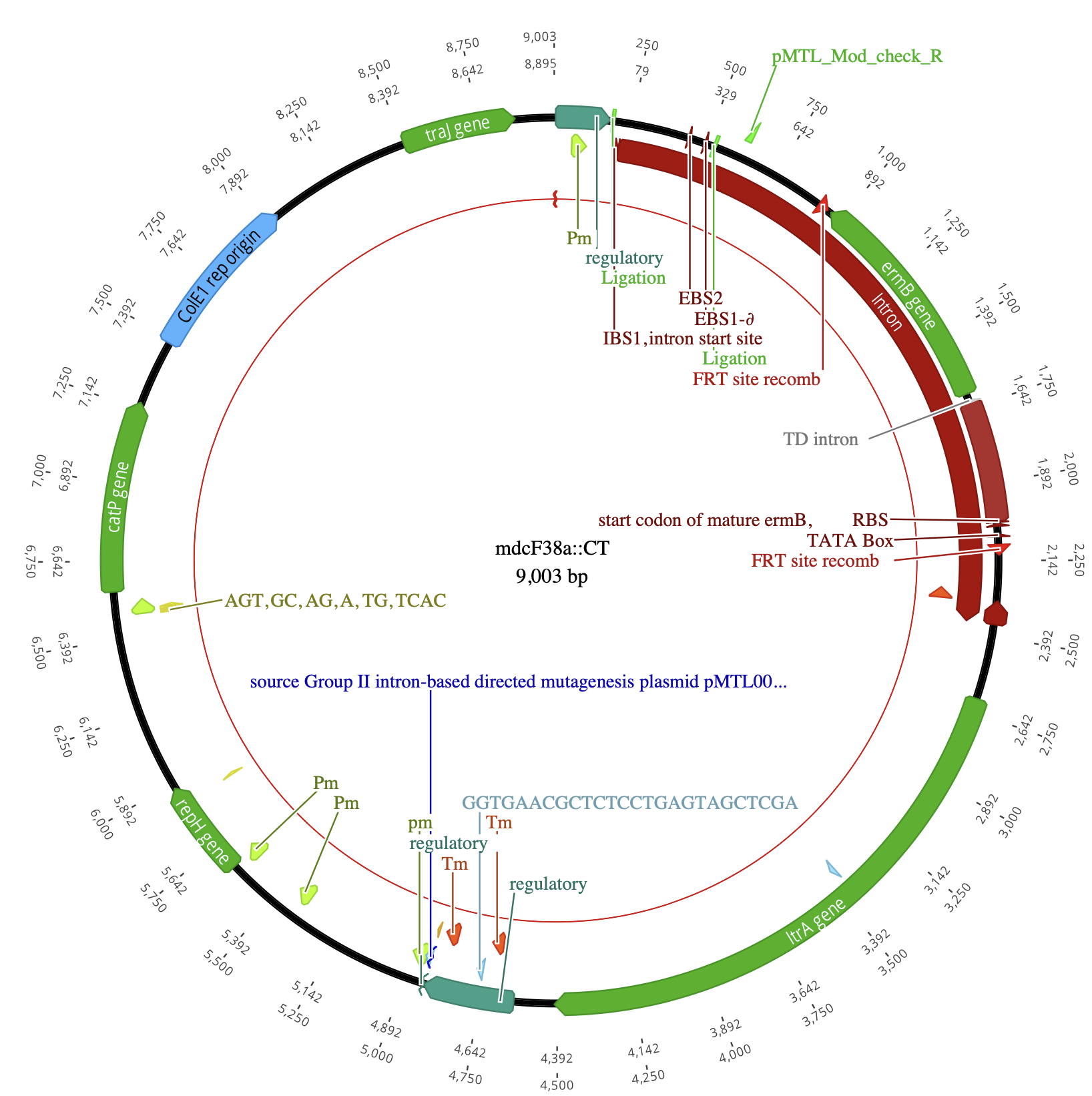
**

**Fig. S5.** Plasmid map of pmH-Mgn-mdcF used in ClosTron method to construct the *M. gnavus* RJX1121 mediterrocin disruption mutant Mgn-RJX1121-mdcF38a::CT.


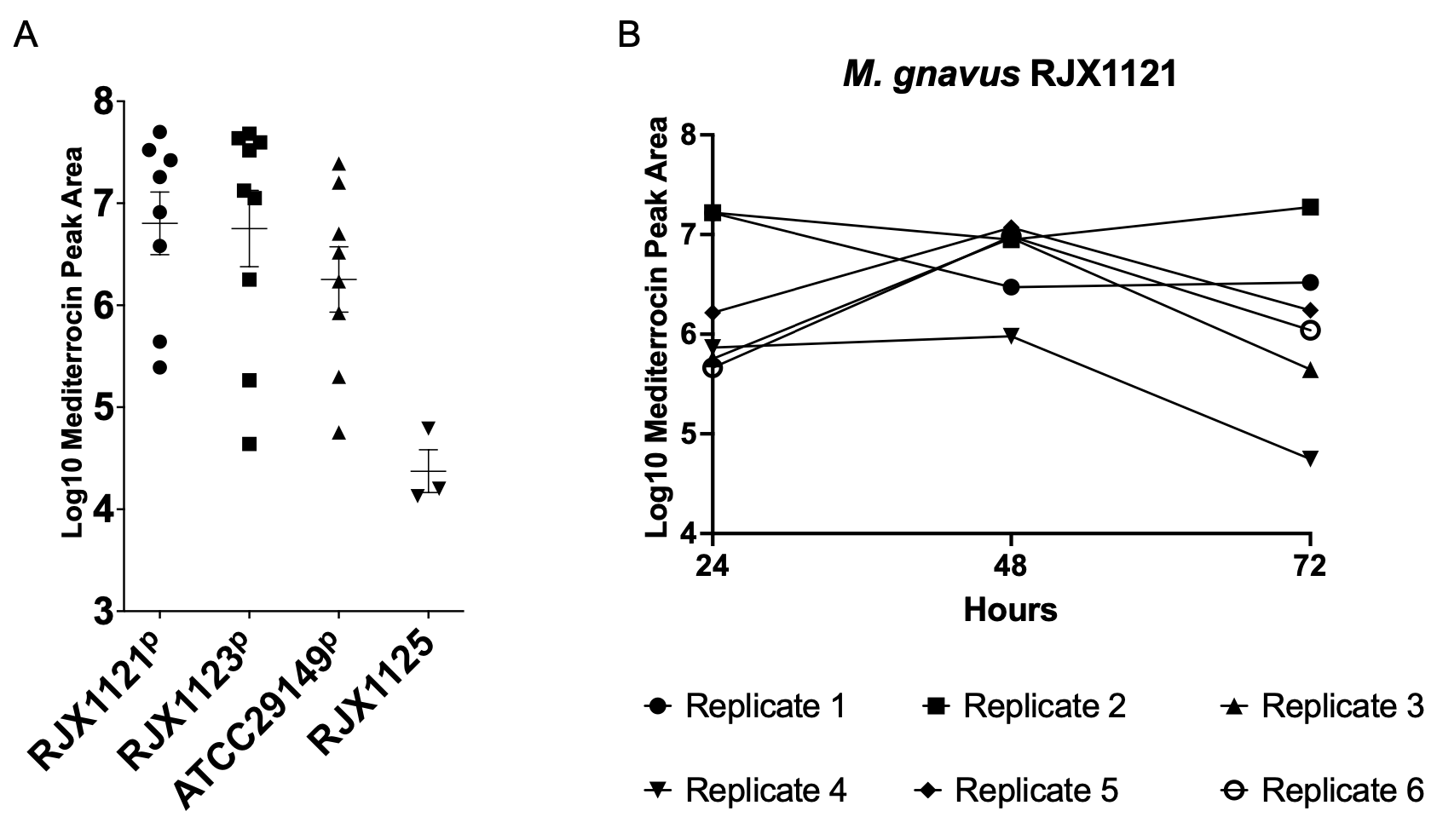


**Fig. S6. (A)** LC-MS/MS quantitation of mediterrocin expression in *M. gnavus* strains RJX1121 (n=8), RJX1123 (n=9), and ATCC29149 (n=8) at 24 hours of growth. Integrated peak areas were determined from the XICs of *m/z* 811.145 ±5 ppm corresponding to the (M+12H)^12+^ charge state of mediterrocin and are represented on a log10 scale. *M. gnavus* RJX1125 does not contain the mediterrocin gene and is shown for reference of baseline noise of *m/z* 811.145. Error bars represent SEM. Superscript p identifies mediterrocin producer strains. **(B)** LC-MS/MS quantitation of mediterrocin expression in *M. gnavus* strain RJX1121 CFS (n=3=6) over 72 hours of growth. Integrated peak areas were determined from the XICs of *m/z* 811.145 ±5 ppm corresponding to the (M+12H)^12+^ charge state of mediterrocin and are represented on a log10 scale.


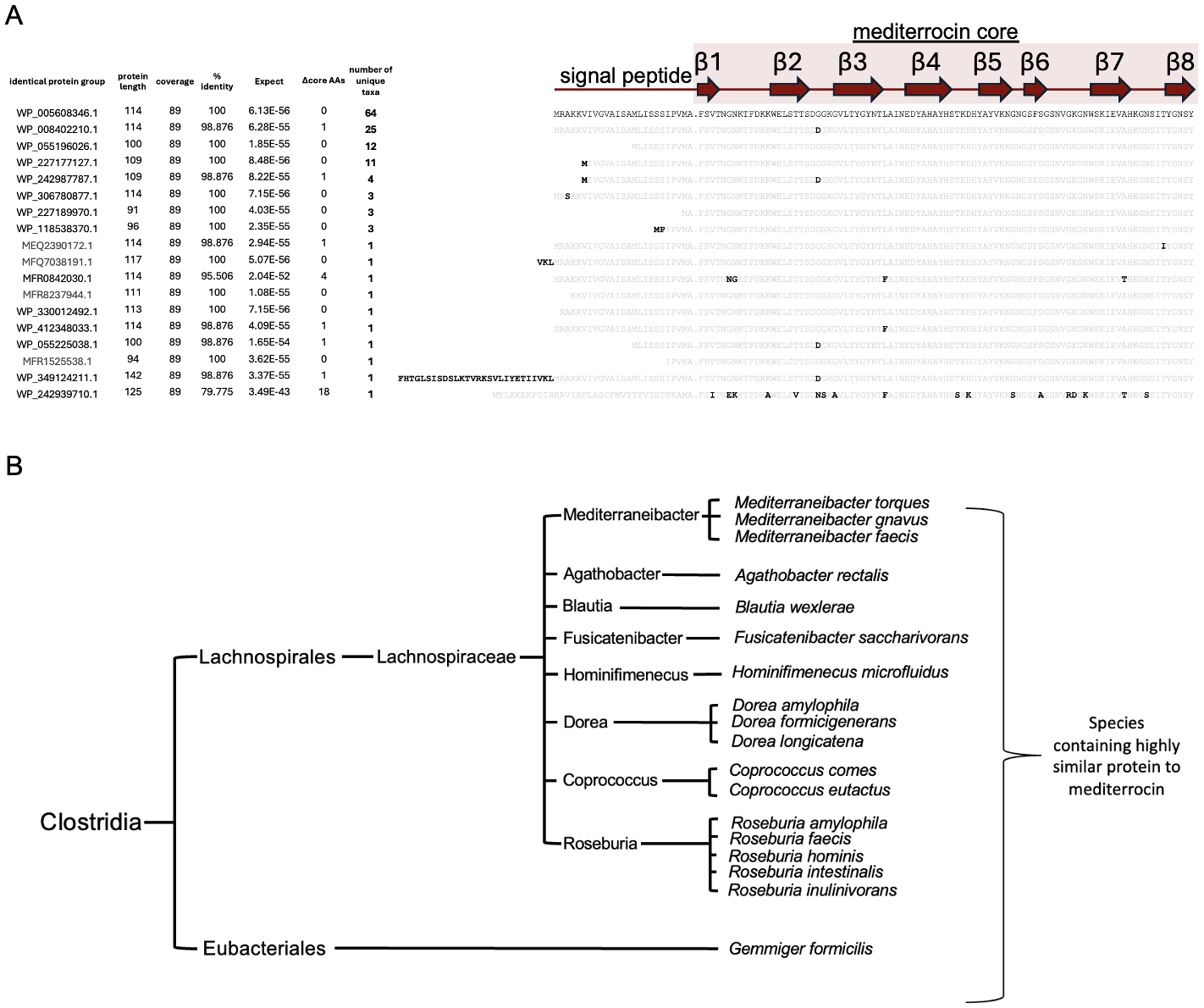
**Fig. S7. Conservation of proteins highly similar to mediterrocin. (A)** Alignment of protein sequences highly similar to mediterrocin in NCBI, defined as 100% coverage and greater than 80% sequence identity to mediterrocin core. The bottom entry represents the drop in identity from 95.5% to 79.7%, which falls outside our definition of highly similar proteins. Mutation sites in the signal peptide or core are highlighted. The number of unique taxa which encode the protein sequence are given. The mediterrocin core is strongly conserved, with only 6 mutation sites in the 89 residue core. The predicted secondary structure of mediterrocin is given above to highlight the position of all mutation sites on loops between β strands. **(B)** Taxonomic tree of species in which at least one strain contains a highly similar protein to mediterrocin, defined above. The bacteriocin homologs are distributed among Clostridia and concentrated within Lachnospiraceae.


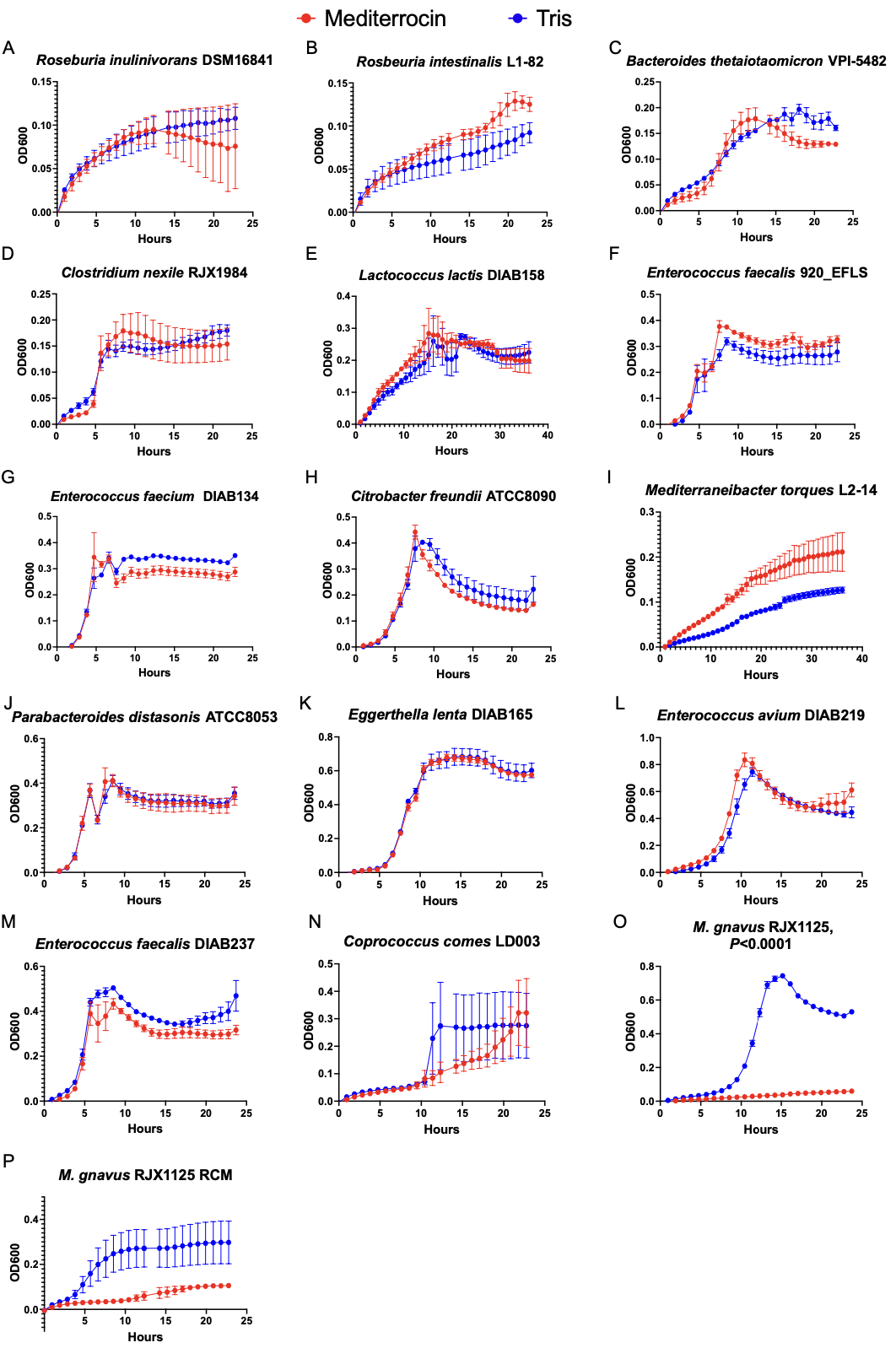


**Fig. S8.** Growth curves of 16 mediterrocin insensitive bacterial strains treated with either 0.75 µg/mL mediterrocin or 20 mM Tris buffer. Sensitive strains were determined from a significant decrease in AUC of mediterrocin treatment compared to control where significance was defined as *p*<0.05. The growth curves of mediterrocin sensitive *M. gnavus* RJX1125 in DM and RCM are shown for reference. *Roseburia* sp., *C. comes,* and *B. thetaiotaomicron* were grown in reinforced clostridial medium (RCM), while all other strains were grown in defined medium (DM). Error bars represent SEM.


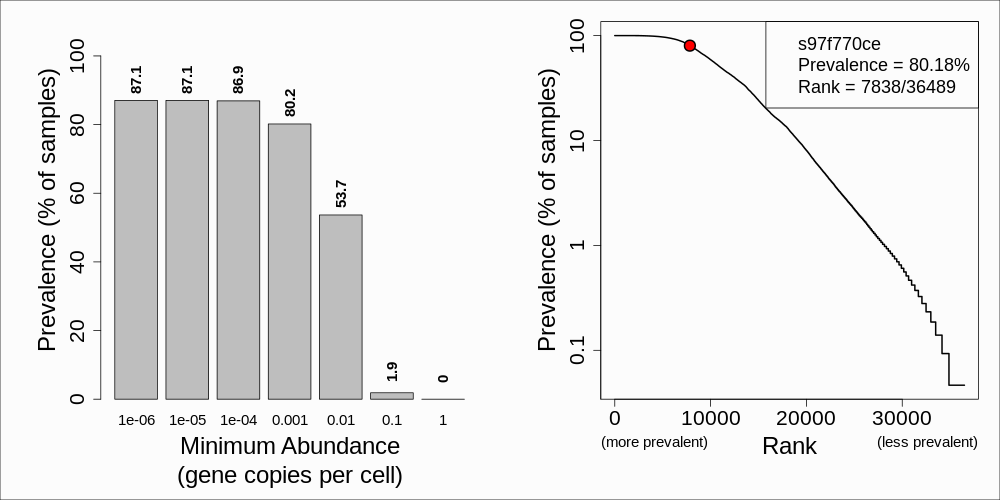


**Fig. S9.** MetaQuery output of mediterrocin prevalence in human gut metagenomes. Parameters used for search: minimum percent identity: 98, maximum E-value: 1e-5, minimum query alignment coverage: 90, and minimum target alignment coverage: 90. Left panel shows the prevalence of mediterrocin from abundance levels of 1E-06 to 1 and the right panel compares the prevalence of mediterrocin to other genes.

**Table S1.** *Mediterraneibacter gnavus* inter-strain competition screen. Cell-free supernatant (CFS) of each *M. gnavus* strain was screened against the indicator strains and OD600 was recorded after 16 hours. Percent growth of the indicator strain was calculated from the ratio of OD600 of treated cultures to untreated culture.The hits from RJX1121 CFS are highlighted in yellow.

|  |  |  | **Producer Strain** | | | | | | | | | |
| --- | --- | --- | --- | --- | --- | --- | --- | --- | --- | --- | --- | --- |
|  |  | **Untreated OD600=100%** | **RJX1119** | **RJX1120** | **RJX1121** | **RJX1122** | **RJX1124** | **RJX1125** | **RJX1126** | **RJX1128** | **ATCC29149** | **ATCC35913** |
| **Indicator Strain % Growth** | **RJX1119** | 0.324 | 26.0 | 64.8 | 25.0 | 31.8 | 86.1 | 110.8 | 42.6 | 40.5 | 44.2 | 51.3 |
|  | **RJX1120** | 0.263 | 23.9 | 109.7 | 134.4 | 62.6 | 107.8 | 135.5 | 107.5 | 32.3 | 80.9 | 66.4 |
|  | **RJX1121** | 0.716 | 124.3 | 121.0 | 114.0 | 134.0 | 148.6 | 142.5 | 146.7 | 116.5 | 100.0 | 134.5 |
|  | **RJX1122** | 0.693 | 100.1 | 91.0 | 73.3 | 82.1 | 76.2 | 95.8 | 98.0 | 142.0 | 68.5 | 72.0 |
|  | **RJX1124** | 0.499 | 44.9 | 23.4 | -0.2 | 20.8 | 34.5 | 70.9 | 37.5 | 26.5 | 67.3 | 72.1 |
|  | **RJX1125** | 0.476 | 37.7 | 41.5 | 50.7 | 55.6 | 65.4 | 74.3 | 53.2 | 57.2 | 49.0 | 48.8 |
|  | **RJX1126** | 0.774 | 123.8 | 88.4 | 68.4 | 94.6 | 107.0 | 109.3 | 111.4 | 117.0 | 91.8 | 84.0 |
|  | **RJX1128** | 0.663 | 43.8 | 9.4 | 0.1 | 60.4 | 76.4 | 87.0 | 45.8 | 43.8 | 74.0 | 71.0 |
|  | **ATCC29149** | 0.703 | 84.6 | 79.8 | 60.9 | 56.0 | 66.7 | 88.4 | 83.2 | 65.3 | 82.6 | 96.3 |

*Supplied as an excel sheet*

**Table S2.** Peptide spectral matching results identified lactococcin 972 family bacteriocin in a partially purified fraction of mediterrocin following SAX.

*Supplied as an excel sheet*

**Table S3.** Proteins identified by peptide-spectral matching including lactococcin 972 family bacteriocin in a partially purified fraction of mediterrocin following SAX.

*Supplied as an excel sheet*

**Table S4-7.** pFind filtered spectra output for each mediterrocin tryptic peptide annotated by MetaPep workflow in four cohorts (PRIDE accessions: PXD007819, PXD008675, PXD011515, and PXD008870).

*Supplied as an excel sheet*

**Table S8.** Mediterrocin tryptic peptides annotated by MetaPep in subjects from metaproteomics datasets (PRIDE accessions: PXD007819, PXD008675, PXD011515, and PXD008870) generated from feces and GI tract sampling. For each subject, the protein sequence coverage, percent identity to mediterrocin, and range of pFind scores are given.

**Supplementary Files**

*Supplied as a text file.*

**SI File 1.** Plasmid sequence of pmH-Mgn-mdcF used to generate mediterrocin disruption mutant *M. gnavus* RJX1121 mdcF38a::CT with the ClosTron method. Fasta sequence.

*Supplied as a text file.*

**SI File 2.** Whole genome assembly of mediterrocin disruption mutant *M*. *gnavus* RJX1121 mdcF38a::CT. Fasta sequence of 1 contig (3.5 Mbp) assembled by Plasmidsaurus. The gDNA was sequenced by Plasmidsaurus using long-read Oxford Nanopore sequencing.
